# The Effects of COVID-19 Lockdown 1.0 on Working Patterns, Income, and Wellbeing Among Performing Arts Professionals in the United Kingdom (April–June 2020)

**DOI:** 10.3389/fpsyg.2020.594086

**Published:** 2021-02-10

**Authors:** Neta Spiro, Rosie Perkins, Sasha Kaye, Urszula Tymoszuk, Adele Mason-Bertrand, Isabelle Cossette, Solange Glasser, Aaron Williamon

**Affiliations:** ^1^Centre for Performance Science, Royal College of Music, London, United Kingdom; ^2^Faculty of Medicine, Imperial College London, London, United Kingdom; ^3^Schulich School of Music, Centre for Interdisciplinary Research in Music Media and Technology, McGill University, Montreal, QC, Canada; ^4^Melbourne Conservatorium of Music, The University of Melbourne, Melbourne, VIC, Australia

**Keywords:** COVID-19, depression, loneliness, wellbeing, work, social connection, performing arts, survey

## Abstract

This article reports data collected from 385 performing arts professionals using the *HEartS Professional Survey* during the COVID-19 Lockdown 1.0 in the United Kingdom. Study 1 examined characteristics of performing arts professionals’ work and health, and investigated how these relate to standardized measures of wellbeing. Study 2 examined the effects of the lockdown on work and wellbeing in the respondents’ own words. Findings from Study 1 indicate a substantial reduction in work and income. 53% reported financial hardship, 85% reported increased anxiety, and 63% reported being lonelier than before the crisis. 61% sought support on finances while only 45% did so on health and wellbeing. Multiple regression analyses, using the Mental Health Continuum-Short Form, Center for Epidemiologic Studies Depression Scale, Social Connectedness Scale, and Three-Item Loneliness Scale as outcome variables, indicate that perceived financial hardship was associated with lower wellbeing and higher depression and loneliness scores. Higher self-rated health was associated with higher wellbeing and lower depression scores. More physical activity before lockdown was associated with higher wellbeing and social connectedness scores, as well as lower loneliness scores, and an increase in physical activity during lockdown compared with before, as well as older age, were associated with higher wellbeing and social connectedness scores, and lower depression and loneliness scores. Thematic inductive analysis of 341 open responses in Study 2 identified five overarching themes characterizing the effects of Lockdown 1.0: *lost or uncertain work and income*, including canceled work, financial concerns, and uncertainties for the future; *constraints of lockdown working*, including challenges of working at home, struggles with online work and skill maintenance, and caring responsibilities; *loss and vulnerability*, including reduced social connections, lack of support, vulnerability, feelings of loss and grief, and concern for others; *detrimental effects on health and wellbeing*, including anxiety, low or unstable mood, poorer physical health, and lack of motivation; and *professional and personal opportunities*, including coping well or living more healthily, more time and less pressure, new possibilities and activities, enhanced social connections, and new skills. Lockdown 1.0 had profound effects on performing arts professionals, but our findings reveal some opportunities and compelling links between positive wellbeing and physical activity.

## Introduction

In 2019, the arts and cultural sector contributed £10.8billion to the United Kingdom (UK) economy and provided employment for 363,700 people (Centre for Economics and Business Research, 2019). Engagement in the arts and culture, in addition to being valued in and of itself (Arts Council England, 2014), has been shown to support health and wellbeing (Fancourt and Finn, 2019). Despite its importance, however, occupational challenges in the sector have been long acknowledged in the UK and beyond (van Liemt, 2014). For instance, performing arts professionals earn a wide range of income (Trends Business Research, 2018), and there are problems with job insecurity and unpredictability (Pasikowska-Schnass, 2019). There are also potentially detrimental health and wellbeing outcomes connected to the work itself or the conditions of work (Wynn Parry, 2004; Tuisku et al., 2016). For instance, artists can experience high work demands and low social support (Holst et al., 2012), insecurity and short-lived careers (Cahalan and O’Sullivan, 2013), overworking (Teague and Smith, 2015), and complex relationships and self-criticism (Robb et al., 2018). More generally, occupational demands have been linked with poorer wellbeing among performing artists (Willis et al., 2019).

Psychological challenges are diverse and include anxiety and depression in, for example, orchestral musicians (Kenny et al., 2014). Vaag et al. (2016) report higher prevalence of psychological distress among musicians than other workers, and actors report a range of pernicious wellbeing threats (Robb et al., 2018). Potentially harmful perceptions, attitudes, and behaviors toward health and some aspects of fitness have also been identified in musicians (Araújo et al., 2017, 2020). Additionally, workers in the performing arts face the risk of injury and performance-related pain evidenced, for example, among circus artists (Shrier and Hallé, 2011), dancers (Jacobs et al., 2017), and musicians (Cruder et al., 2018). Issues connected with wellbeing and depression are also associated with physical injury and performance-related pain, as well as with the brevity and insecurity of careers, such as in the case of dancers (Cahalan and O’Sullivan, 2013).

Social support seems to be a key contributor to sustaining wellbeing among those working in the performing arts. For example, low levels of social support have been associated with high stress levels in orchestral musicians (Holst et al., 2012). Notwithstanding the portfolio nature of many performing arts professionals’ work (Bennett, 2009) – with irregular or flexible hours and engagement with different groups of people for varying amounts of time – social aspects of work, such as making music and sharing musical moments, have been observed to contribute to wellbeing in the case of musicians (Ascenso et al., 2017) and of dancers, who value the friendships they develop (Cahalan and O’Sullivan, 2013). Despite the majority of research focusing on musicians’ ill-health, studies that focus on wellbeing in terms of positive functioning show that musicians have promising profiles (Ascenso et al., 2018). More generally, jobs in the performing arts sector have been associated with higher levels of subjective wellbeing than ‘non-creative’ jobs (Fujiwara and Lawton, 2016).

The COVID-19 pandemic and its associated lockdowns have had negative impacts on health and wellbeing. Brooks et al. (2020), in a rapid review of the evidence, report that quarantine can lead to negative psychological effects including confusion and anger, with stressors including boredom, inadequate information, and financial loss. In an analysis of data from 36,520 adults in the UK, Fancourt et al. (2021) showed that 22% had scores indicating moderate-severe anxiety, and 26% had scores indicating moderate-severe symptoms of depression at the end of March 2020. In another study, Evanoff et al. (2020) found that family or home stressors during the pandemic were associated with poorer mental health outcomes. Finally, Wright et al. (2020) showed that cumulative worries and experience of adversities during the pandemic – such as financial worries or loss of work – are associated with higher levels of both depression and anxiety.

Against this backdrop, the first lockdown in 2020 had multiple implications for those working in the performing arts specifically. In this article, we define performing arts professionals as those who identify as working in sectors where arts are often performed live in front of an audience or onlookers. This includes those working as performers directly as well as those working as educators, administrators, and researchers in this sector. In the UK, performing venues were shut completely for several months, and rehearsals or other forms of artistic engagement, including teaching, were prohibited in person, either stopping entirely or moving online. People’s jobs were furloughed, and many people became unemployed. Furthermore, many performing arts professionals in the UK are self-employed and therefore particularly vulnerable to the cancelation of work. Indeed, in general the creative sector in the UK may be impacted twice as hard as the wider economy, with 406,000 jobs at risk and some sub-sectors losing more than half their revenue and workforce (Oxford Economics, 2020). The social impact has been equally dramatic, with professionals not able to meet and co-create their work in person. Some aspects of the impact of these changes may be immediate and others may be much more long term, so it is important to understand and to document how the initial lockdown in 2020 impacted on key areas of performing arts professionals’ working lives and wellbeing.

This article responds to this need by posing two related research questions:

RQ1. What were the characteristics of performing arts professionals’ work profiles, income, and health during COVID-19 Lockdown 1.0 and how did these relate to measures of their mental and social wellbeing?
RQ2. What were the impacts of COVID-19 Lockdown 1.0 on the work and wellbeing experiences of performing arts professionals, in their own words?

The research questions were addressed in two studies. Study 1 examined RQ1 through a snapshot of respondents’ work and COVID-19-specific characteristics as well as their demographic characteristics. These were then examined in relation to measures of mental and social wellbeing. Study 2 investigated RQ2 by exploring the impact of lockdown as experienced and described by respondents themselves.

## Study 1

### Method

#### Respondents

The survey was open to professionals working in the arts in any capacity (Supplementary Figure 1, questions 4.1 and 4.2). In this article we focus on the subset of respondents who work in the performing arts; those who identify as working in sectors where arts are often performed live in front of an audience or onlookers. We therefore include only those who reported working in at least “music or sound arts” and/or “performing arts” in a range of capacities (including performing, teaching, music therapy, and managing). Demographic characteristics of the 385 performing arts professionals based in the UK are summarized in Supplementary Table 1.

Many of the respondents reported several professional activities. Over two thirds (*n* = 260, 68%) included music or sound arts (with classical music being highly represented, *n* = 155) and over half included performing arts (*n* = 201, 52%) such as acting, dancing, and musical theater. About two thirds of the respondents identified as female (*n* = 242, 63%). Participants were aged 18–86 years. Seventy percent of the respondents were aged between 26 and 55 (*n* = 268, mean age = 44.08, *SD* = 13.9). The majority of respondents were white (*n* = 357, 93%), about half of the respondents had a tertiary degree (*n* = 165, 51%) and London was home to the largest group of respondents (*n* = 162, 42%). Almost a quarter lived with children (*n* = 91, 24%), and 17% lived alone (*n* = 67). Over a third (*n* = 138, 36%) had a household income of more than £52,000, although household income spanned the whole range of the scale (£0-£76,000+). Our respondents contributed to over half of that income (59% on average) with about three-quarters of income coming from work in the arts (77% on average). The majority of respondents had not had or not knowingly had COVID-19 (*n* = 328, 85%).

The majority of respondents positively rated their health (*n* = 330, 86% rating it “Very good” or “Good”). The majority did not report chronic health conditions (*n* = 263, 68%) while half reported a reduction in physical activity in the last month (*n* = 194, 50%) (Supplementary Table 2).

How similar is our sample of participants to performing arts professionals? According to the 2018–2019 Art Council report on equality and diversity in the institutions they support, 60% identified as female, 35% as male, less than 1% as non-binary, 2% preferred not to say, and for 3% gender was not reported (Arts Council England, 2018). Our sample lines up closely with this distribution. In terms of ethnicity, 11% of respondents identified as “Black and Minority Ethnic background” and 53% were White. The ethnicities of the rest were not known. So out of the workers about whom ethnicity is known, 83% were white. In terms of age, the categories used in our study are somewhat different from those in the Arts Council report; however, for broad comparison, 60% of the Arts Council study about whom age is known were 49 years or younger compared with 55% in our study. Although this report does not cover all workers in the arts and cultural sector this comparison suggests that our sample is close to the proportions of the sector on at least some demographic characteristics.

#### Procedure

Data were collected via *HEartS Professional*, a survey of workers in the arts and cultural sectors to determine the impact of the COVID-19 pandemic on their health, wellbeing, and livelihoods. *HEartS Professional* (see Supplementary Figure 1) is an adaptation of the *HEartS Survey* which charts the Health, Economic, and Social impacts of the ARTs (Tymoszuk et al., 2021). *HEartS Professional* was designed as a multi-strategy data collection tool with two main purposes: (1) to chart working patterns, income, sources of support, and indicators of mental and social wellbeing in order to identify trends in the effects of lockdown, and (2) to explore the individual work and wellbeing experiences of performing arts professionals in their own words. The survey therefore covered six areas: (1) demographics, (2) information on illness or self-isolation related to COVID-19, (3) work profiles and income, (4) changes to work profiles and income as a result of the pandemic, as well as sources of support, (5) open questions about work and wellbeing experiences of lockdown including challenges and opportunities, and (6) validated measures of health, wellbeing, and social connectedness. Study 1 reports on areas 1–4 and 6 of *HEartS Professional* in order to chart working patterns, income, sources of support, and indicators of mental and social wellbeing and to identify trends in the effects of lockdown on performing arts professionals.

The Conservatoires UK Research Ethics Committee granted approval on March 31, 2020. Informed consent was obtained at the start of the online survey. The intention was to recruit as broad a spectrum of people as possible who were over the age of 18 years, working in the arts and cultural sector, and living across the UK. Our recruitment process was inspired by Respondent Driven Sampling (RDS) methods. Although we did not carry out the systematic analyses of participants’ social networks that would be required for a comprehensive application of RDS, we sent direct email invitations and tagged tweets to “seed” participants who belonged to the arts communities relevant to our project (Heckathorn and Jeffri, 2003; Gile et al., 2015). Recruits were invited to forward the email invitation or tweets to others they thought may be interested. We targeted cultural and arts organizations as well as individuals. In order to be as comprehensive as possible, we reached out to the higher education institutions that include degrees in the arts (UCAS, 2020) and nationwide arts organizations (e.g., the Incorporated Society of Musicians). We also reached out to major arts companies (e.g., the BBC Philharmonic) and venues (e.g., The Globe Theater). Finally, given that so many performing arts professionals work freelance and are not likely to be actively connected to their former educational institutions, nationwide organizations, or arts venues and companies, we also reached out to individuals – chosen either because they are well-known artists or because they were in our professional networks. Although we aimed for overlap between email and twitter, sometimes this was not possible either because appropriate points of contact were not obvious or because twitter accounts were not available. Finally, we experimented with recruitment through paid Facebook adverts which were live between April 21 and May 21, 2020. The link on the advert was clicked 55 times.

Data reported here are cross-sectional and based on a subset of the data focusing on performing arts professionals living in the UK. The survey was open for responses from April 01 to June 15, 2020, and almost half of the respondents completed the survey in the first two weeks of April (*n* = 189, 49%). A copy of the dataset is publicly available (Williamon et al., 2021). The same survey was simultaneously launched and closed in four more countries (Australia, Canada, New Zealand, and United States) with local adaptations made to some demographic questions, such as the geographic areas in which people lived and the currencies used for reporting income.

#### Outcome Measures

Led by the view that both positive- and ill-health contribute to how we experience our mental and social wellbeing (Seligman, 2008), we used one outcome measure each for a positive view and symptom-led view of mental and social wellbeing. More specifically, for mental wellbeing we focused on mental health and depression, and for social wellbeing, we focused on social connectedness and loneliness. For mental wellbeing we used the 14-item Mental Health Continuum – Short Form [MHC-SF; Keyes, 2002, 2005 which had Cronbach’s alpha (α) of 0.91 for the subset of the data used in the regressions]. The MHC-SF measures hedonic dimensions of wellbeing (3 items), as well as eudaimonic dimensions (11 items). Each of the 14 items is rated on a 6-point scale (0 “never,” 1 “once or twice,” 2 “about once a week,” 3 “2 or 3 times a week,” 4 “almost every day,” 5 “every day”) generating a continuous score ranging from 0 to 70. In addition, a categorical variable can be derived denoting: “flourishing mental health” (for respondents experiencing at least one of the three hedonic dimensions and at least six of the eleven eudaimonic dimensions “every day” or “almost every day”), “languishing mental health” (for respondents experiencing at least one of the three hedonic dimensions and at least 6 of the 11 eudaimonic dimensions “never” or “once or twice” in the past month), and “moderate mental health” (for respondents in between the two previous categories). For depression, we used the eight-item Center for Epidemiologic Studies Depression Scale (CES-D, α = 0.76). Each of the eight items has Yes or No response options, and the number of present depressive symptoms is summed generating a score ranging from 0 to 8. A score of three symptoms or more (out of a possible eight) has been commonly used for identifying cases of depression (Karim et al., 2015). For social connectedness we used the 15-item Social Connectedness Scale (Lee et al., 2008, α = 0.69), where a higher score indicates more connectedness to others. For loneliness, we used the Three-Item Loneliness Scale (Hughes et al., 2004, adapted from the Revised UCLA Loneliness Scale, Russell et al., 1980), which identifies those scoring 6 or higher out of a possible 9 as lonely (α = 0.76).

#### Analysis

Of the 744 people who completed the consent section of the survey, 449 reached the final question. For this article, we include only those who chose at least one performing arts area (“music or sound arts” and/or “performing arts,” Supplementary Figure 1, question 4.1). This left 389 participants. All of the closed questions (i.e., all those in sections 1–4 and 6) were compulsory. We did, however, exclude cases from the dataset if there was evidence of response bias such as straight-lining (*n* = 3) or extreme responses (*n* = 1). This left us with a sample of 385 respondents.

Data were analyzed using descriptive statistics to provide an overview of general patterns of work, income, perceived changes in anxiety and loneliness, and support sought. We created a correlation matrix to explore the relationships between the four outcome variables. Tests for normality of distribution showed that the total scores for depression (CES-D) and loneliness (Three Item Loneliness Scale) showed significant deviations from normality (Kolmogorov–Smirnov *p* < 0.05), so Spearman’s rho inter-correlations were calculated.

We ran four separate hierarchical multiple linear regression models using SPSS (v.25) to explore the relationship between COVID-19-related, demographic, and arts work variables and the levels of mental wellbeing, depression (following McHugh and Lawlor, 2012), social connectedness, and loneliness (following Rico-Uribe et al., 2016; Ge et al., 2017).

In all four regressions, Model 1 was adjusted for four pandemic-specific variables (Supplementary Tables 5A–D): timeframe of survey completion (Timeframe), physical activity during the pandemic (Lockdown exercise), perceptions of financial hardship (Financial hardship), and changes in socializing with others, both online and in-person (Socializing change). Timeframe of survey completion was operationalized as a binary variable; respondents were dummy coded as “1” if they completed the survey just as the lockdown measures were being imposed – within the first two weeks of the survey’s distribution – or “0” if they completed the survey later – after the first two weeks. Changes in physical activity during lockdown were coded on a continuous scale from −3 to 3, where “much less physical activity,” “quite a bit less physical activity,” and a “little less physical activity” were assigned negative values ranging from −3 to −1. No change in physical activity was coded as “0,” and “a little more physical activity,” “quite a bit more physical activity,” and “much more physical activity” were assigned values ranging from 1 to 3. Perceptions of financial hardship were operationalized as a dummy variable, with respondents who responded to “Do you consider yourself to be in financial hardship as a result of the current public health situation?” with “yes, a little” or “yes, a lot” coded as “1,” and respondents who responded “No” coded as “0.” Changes in socializing patterns overall were calculated by averaging self-reported change in socializing with others in-person and online.

In addition to these variables, Model 2 adjusted for covariates related to demographic and work characteristics. Variables associated with demographic factors and arts work were: gender, age, ethnicity, living status (Living alone), self-rated health (Health), exercise habits prior to COVID-19 (Pre-COVID-19 exercise), educational attainment (Ed. attainment), art specialism, household income (Household income, which includes all earnings including for example, from pensions), percentage of time spent freelancing (% freelance), individual contribution to household income (% Cont. income), and the percentage of one’s individual contribution to household income generated from arts work specifically (% Cont. art). Gender was operationalized as a binary variable. To move beyond the traditional, and inadequate, binary view of gender (Hyde et al., 2019) and step toward a more inclusive approach to gender, we do not exclude non-binary respondents. There is no standard measure of gender identity in quantitative research (Fraser, 2018), but one option proposed by Fraser (2018) is to dummy code gender identity as is often done with ethnicity data. Here, we group women and non-binary respondents – as has been done in queer and feminist approaches to social science interventions (Kos, 2019) – to create a binary variable. We dummy code respondents who self-identified as female or chose “other” as “0” and those who self-identified as male “1”. Ethnicity was operationalized as a binary variable (white = 1, non-white = 0), as were living status (living alone = 1, other living configurations = 0), and educational attainment (university degree or equivalent = 1, other qualifications = 0). The remaining variables were entered as continuous variables. Participants’ ages were represented in whole numbers (e.g., a 20-year-old respondent would have an age score of “20”). While household income, self-rated health, and exercise habits prior to COVID-19 are ordinal variables, they were treated as continuous variables based on the practice that ordinal variables with five or more categories can be treated as continuous variables without risking the quality of the analysis.

Ordinary least squares regression assumptions were checked: the assumption of normality of residuals was tested using kernel density plot, standardized normal probability (P–P) plot, and quantile (Q–Q) plots, while homoscedasticity of residuals was established by plotting residuals versus predicted values. The four regression models were conducted using a smaller subset of 353 participants; participants were excluded from regression analyses if they answered “prefer not to answer” for any survey questions, or if cases were outliers as determined by residual *z*-scores, Mahalanobis distance, Cook’s distance, and leverage statistics (*n* = 28). No issues of multicollinearity were identified.

### Results

#### Changes in Work Profiles

**FIGURE 1:**
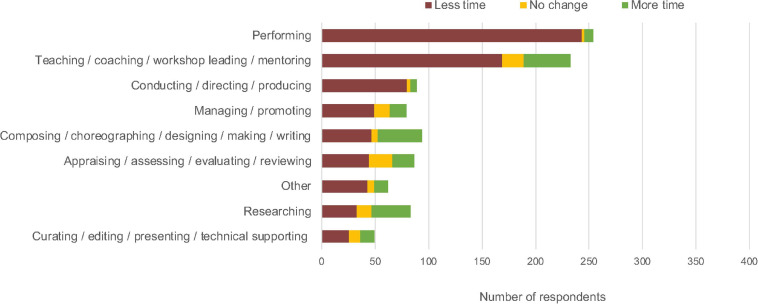
Change in time spent working for all respondents, from April–June 2020 compared with before Lockdown 1.0 (respondents were asked to select all areas of work that applied to them).

Overall, time spent working reduced (Figure 1 and Supplementary Table 3A). In areas of work most affected, an overwhelming majority of respondents reported a reduction in working time: 96% (*n* = 243) reported less time spent performing (with 87.4% of respondents reporting *much less time* spent, *n* = 222), 90% (*n* = 80) reported less time *conducting/directing/producing*, and 73% (*n* = 169) reported less time *teaching/coaching/workshop leading/mentoring*. The other areas of work spanned the range in between. For example, 50% (*n* = 47) reported less time spent *composing/choreographing/designing/making/writing*, while 62% (*n* = 49) saw a reduction in *managing/promoting*. Averaging across all areas of work, 71% of respondents spent less time working than before.

Areas of work that saw the largest reductions in time spent were predominantly offline and with others (Supplementary Table 3B). For example, 89% of performers typically performed offline and with others before COVID-19, whereas offline with others subsequently dropped to 7% and was not replaced with a similar increase in online interactions with others, which went up only to 21%. However, the patterns are not straightforward. For example, *appraising/assessing/evaluating/reviewing* moved more decisively from 77% offline (52% with others and 24% alone) to 79% online (34% with others and 45% alone), with half of these respondents also experiencing a reduction in their working hours. *Teaching/coaching/workshop leading/mentoring* saw a move online (95% offline pre-COVID-19 to 83% online), but the time spent working in this area dropped for 73% of respondents (Supplementary Table 3A).

**FIGURE 2:**
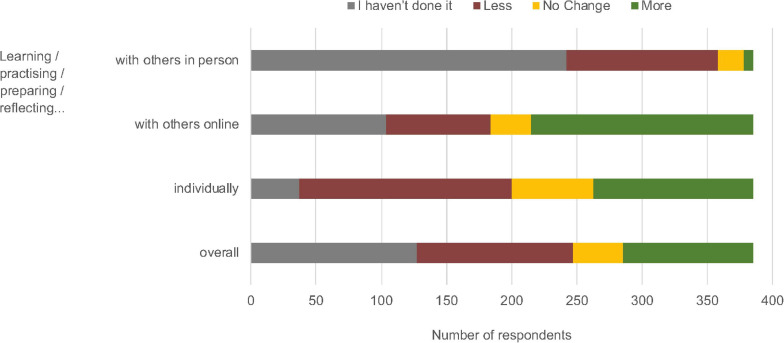
Changes in the maintenance and development of skills for all respondents, from April–June 2020 compared with before Lockdown 1.0.

Changes in skill maintenance and development were investigated using the question “Overall, since the start of the current public health situation, how often you have been able to maintain your skills as an artist, performer, maker etc.?” (Supplementary Table 3C). As shown in Figure 2, 61% reported not *learning/practicing/preparing/reflecting* with others in person since the start of the pandemic, and a further 30% reported doing so less. Additionally, about half of our respondents were doing fewer individual activities associated with skill maintenance and development (42% reported *learning/practicing/preparing/reflecting* individually less than before, and 10% had not done it at all). Overall, 33% of respondents indicated they had not engaged with *learning/practicing/preparing/reflecting* in any medium, and 31% of respondents had engaged with these activities less overall since the start of the lockdown (see Figure 2).

The financial implications seemed clear early on: by mid-June 2020, 76% of respondents had experienced a decrease in income – nearly 30% reported having already lost over £4000 – and 54% considered themselves to be in financial hardship (Supplementary Tables 3E,F).

#### Changes in Loneliness and Anxiety

**FIGURE 3:**
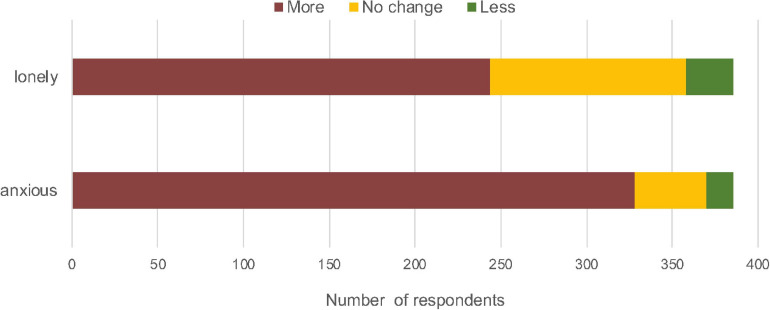
Changes in loneliness and anxiety over the last month for all respondents, from April–June 2020 compared with before Lockdown 1.0.

There was a substantial increase in social meetings online: 88% (*n* = 340) reported an increase in online social meetings, accompanying a 95% (*n* = 366) decrease in in-person social meetings (Supplementary Table 3D). Despite this, there is an overall drop in socializing, with 70% (*n* = 270) of respondents socializing with fewer people. To explore feelings of change in loneliness and anxiety during this period, participants responded to two 1-item questions “In the last month, how has the public health situation affected how [lonely/anxious] you feel?” on a 7-point scale (from Much more lonely/anxious to Much less lonely/anxious), here grouped into three categories (More, No change, and Less). 63% (*n* = 244) reported feeling more lonely, and 85% (*n* = 328) reported feeling more anxious as a result of the public health situation (Figure 3 and Supplementary Table 3G).

#### Mental and Social Wellbeing and Their Associated Variables

According to the classification of scores in the MHC-SF scale, over half the participants reported “moderate” levels of wellbeing (55%), whereas over a third were “flourishing” (34%), while only 11% were “languishing” on the 14-item MHC-SF scale. Over two thirds of the sample (69%) reported three or more depressive symptoms on the 8-item CES-D scale, and hence could be described as depressed (Karim et al., 2015; Supplementary Table 4); the mean score was 4.03 (out of 8; *SD* = 2.35). The average score for social connectedness was 48.56 (*SD* = 12.41) on the 15-item Social Connectedness Scale-Revised which has a range of 0–75, which is close to the mean found in our previous work with a broader population before COVID-19, where the mean was 41.48 (*SD* = 15.49) (Tymoszuk et al., 2021). Forty-one percent (*n* = 157) of the respondents scored 6 or higher out of a possible 9 on the Three-Item Loneliness Scale (Hughes et al., 2004, adapted from the Revised UCLA Loneliness Scale, Russell et al., 1980) and were therefore classed as lonely (Steptoe et al., 2013), with an average score of 5.12 (*SD* = 1.66). The correlations between all four outcome measures (Supplementary Table 4) were in the low to moderate range (Mukaka, 2012).

With this overview of the outcome measures in mind, we explored these findings using regression analyses to investigate which factors impacted on these outcomes. Model 1, adjusting for the COVID-19 related factors only, predicted between 4 and 7% of the variance in the outcomes (Supplementary Tables 5A–D). The fully-adjusted model, which included also demographic and work characteristics (Model 2), explained 13% of the variance in wellbeing (MHC-SF, adjusted *R*^2^ = 0.131, *F*_16_,_352_ = 4.32, *p* < 0.001), 12% of the variance in depression (CES-D, adjusted *R*^2^ = 0.117, *F*_16_,_352_ = 4.76, *p* < 0.001), 12% of the variance in social connectedness (Social connectedness scale, adjusted *R*^2^ = 0.119, *F*_16_,_352_ = 3.97, *p* < 0.001), and 15.3% of the variance in loneliness (Three-Item Loneliness Scale, adjusted *R*^2^ = 0.153, *F*_14_,_352_ = 4.97, *p* < 0.001).

Age was associated with all four outcomes measures. Age was positively associated with wellbeing (*B* = 0.21, *p* < 0.001) and social connectedness (*B* = 0.17, *p* < *0.001*), and negatively with depression (*B* = −0.05, *p* < 0.001) and loneliness (*B* = −0.02, *p* = 0.001); in other words, as age increased on the linear scale, wellbeing and social connectedness scores increased while depression and loneliness decreased.

There were associations between physical activity and the outcome measures. Physical activity during lockdown (Lockdown exercise, Supplementary Tables 5A–D) was associated with better outcomes across all models. Physical activity during lockdown was associated with higher wellbeing scores (*B* = 1.25, *p* < 0.001) and social connectedness scores (*B* = 0.84, *p* = 0.003), and with lower loneliness (*B* = −0.12, *p* = 0.007) and depression scores (*B* = −0.16, *p* = 0.010). In addition, physical activity prior to COVID-19 (Pre-COVID-19 exercise) was associated with higher wellbeing scores (*B* = 0.64, *p* = 0.008), higher social connectedness scores (*B* = 0.51, *p* = 0.008), and lower loneliness scores (*B* = −0.07, *p* = *0.026*). Self-rated health (Health) was associated with higher wellbeing scores (*B* = 2.54, *p* = 0.011), as well as lower depression scores (*B* = −0.41, *p* = 0.019).

We also saw associations between when the survey was completed (Timeframe) and three of four outcome measures. Early completion was associated with better outcomes than later completion: in the covariate-adjusted models, completion within the first two weeks was associated with higher wellbeing (*B* = 4.42, *p* = 0.002) and higher social connectedness scores (*B* = 2.89, *p* = 0.012) and loneliness scores (*B* = −0.39, *p* = 0.027). Timeframe of completion was not a meaningful exposure variable associated with depression, however (*p* = 0.052).

Perceived financial hardship was associated with worse outcomes in both of the mental wellbeing models and one of the social wellbeing ones. Perceived financial hardship was associated with lower wellbeing scores (*B* = −1.28, *p* = 0.006), and higher depression and loneliness scores (*B* = 0.18, *p* = 0.030; *B* = 0.13, *p* = 0.024).

In addition to the common variables across analyses, there were also variables relevant to only one or two regression analyses. Those living alone had higher loneliness scores (*B* = 0.70, *p* = 0.011). Gender emerged as a statistically significant variable associated with depression: male participants had reported lower depression scores compared with women and non-binary respondents (*B* = −0.69, *p* = 0.006). Finally, a higher percentage of freelance work relative to employed work emerged as a significant predictor of loneliness, but this finding’s directional relationship and magnitude should be treated with caution, as its effect is likely small (*B* = −0.01, *p* = *0.041*).

#### Seeking Support

Of the 205 respondents who reported experiencing financial hardship, 124 (61%) sought support on finances, while fewer (*n* = 91, 45%) did so for health and wellbeing (Supplementary Table 6). The two most popular sources of support were colleagues and family/friends, but the order is opposite for the two issues: more turned to colleagues on finances (69%) than on health (59%), while more turned to family or friends on health (66%) than on finances (59%). Trade unions were the most popular formal agencies for financial support (45%), and health professionals were the most popular formal routes for support for health and wellbeing (28%).

### Discussion

The results of the regression analyses suggest that two pandemic-related factors (earlier survey completion and more physical activity during the pandemic) and three demographic factors (better self-rated health, more physical activity pre-COVID-19, and older age) predict better scores on at least one mental wellbeing and at least one social outcome measure. Perceived financial hardship was associated with worse wellbeing and depression. Living alone was associated with higher loneliness scores. Finally, male participants had lower depression scores compared with women and non-binary respondents.

In general, the changes in time spent on different areas of work line up with whether the mode of work lended itself to the current situation (e.g., some forms of research) or not (e.g., performance). However, even research and teaching – activities that are perhaps more conducive to online working – were substantially affected, as were activities such as composing that are not obviously public facing. Reduction in in-person work was, for many, not replaced, despite there being opportunities to move online. A number of factors may be contributing to these findings. The survey was carried out early in the pandemic. An optimistic interpretation is that people were taking time to adapt to online or alternative working patterns. Other contributors could be the context and chain of work that typically surrounds performing arts activities. For example, although the act of composing may occur in isolation, it is often part of a network of activities, including commissioning and public performance. Similarly, while many performing arts can be taught online, for some art forms this is more challenging, and even when it is plausible, technical barriers such as lack of access to the relevant technology or lack of or bad internet connection can interfere. The impact on income was swift and, for many, dramatic: within a matter of weeks respondents experienced loss of income, even in work areas that are typically longer term. With this backdrop, we also see the substantial drop in ability to maintain and develop professional skills, providing further evidence of substantial disruption to working lives.

In terms of the factors associated with social and wellbeing outcomes, the importance of the *timing of survey completion* reflects the rapidly developing situation from April to mid-June 2020 and also suggests that a longitudinal follow-up is essential to trace how this unstable situation changes. The importance of *physical activity* and *self-rated health* identified here is in line with previous research demonstrating the importance of physical health in the performing arts (Cahalan and O’Sullivan, 2013; Donohue et al., 2018; Araújo et al., 2020) and during COVID-19 (Wood et al., 2020). Particularly striking is the implication of the possibility for change; although we do not know the causal relationship, it is interesting that exercise during lockdown was associated with positive outcomes on all measures, suggesting both the importance of this activity and the possible effectiveness of creating new habits.

The importance of *age*, and the association between being younger and having worse outcomes, initially seems surprising. At the time, COVID-19 was thought to be less of a threat to younger people and adaptation to new working and social situations required technological know-how generally considered more available to younger people. However, other factors may be over-riding these COVID-19-specific ones. Older performing arts professionals may be more established in their careers and home lives. In the short term, at least, there may be feelings of enough social security that could lessen the impact on mental and social wellbeing. Older performing arts professionals may also have developed reputations and may therefore be more sought after by pupils, mentees, or collaborators who are willing to invest more to keep working together. Older performing arts professionals may also have older students and collaborators who are themselves invested in continuing to work. Further research is required to understand the reasons for the association between age and mental and social wellbeing outcomes and the ways in which younger performing arts professionals can develop their work in these new contexts and how they can be supported in doing so. Future studies, with bigger data sets, are needed to identify which age categories are most likely to be associated with better or worse mental and social outcomes.

The connections revealed in regression analyses between financial hardship and poor mental health are in line with previous research (Butterworth et al., 2009). Similarly, associations between gender and depression are not newly identified in this study (Voltmer et al., 2012). While there are likely several contributing factors here, one possible explanation may be in the relatively high rate of women’s domestic responsibilities. While women already take on the majority of childcare responsibilities on average (Della Giusta et al., 2011), many women took on the brunt of childcare responsibilities during lockdown, had less time for paid work, and spent more time on unpaid labor (Office for National Statistics (ONS), 2020). Additionally, research has suggested that women are more likely than men to rely on social networks to address depressive symptoms (Martínez-Hernáez et al., 2016) which, at this time when people were meeting less in person, may be particularly pertinent. Future research is needed to trace whether these associations remain.

In terms of support, it is notable that over half of those questioned did not seek help for health and wellbeing even though a majority reported increased anxiety. This finding chimes with previous research that has identified awareness of and access to support as a topic for concern (Williamon and Thompson, 2006; Araújo et al., 2017; Ackermann, 2019). Early in the pandemic in the UK, organizations made information about support available (e.g., British Association for Performing Arts Medicine, 2020; Musicians’ Union, 2020; PPL, 2020; Spotify, 2020). Future research is needed to investigate the reasons why these services were not accessed in the immediate situation, and whether they have been since.

Loss of work, financial difficulties, and mental health issues are not unique to those working in the performing arts at this time (Galea et al., 2020). Taken together with the changes in performing arts work and maintenance of skills, however, the results of this study point to specific needs and areas that require attention. State-determined restrictions and support will play their part. But we already see variability in this study: some people seem to have been able to adapt at least some of their work more quickly than others, some have not felt negative impact on their social and mental wellbeing. Moreover, the situation is not static, and it is unclear what the medium and long-term implications will be – positive or more likely negative – for the performing arts. A longitudinal view is therefore essential in tracing the changes, identifying and sharing innovation and ways of coping, and offering avenues of support.

## Study 2

### Method

#### Respondents

Study 2 reports on a subset of the data analyzed in Study 1, including responses from 341 performing arts professionals based in the UK. Many of the respondents identified multiple professional activities, with 67% (*n* = 227) including music or sound arts in their professional portfolio and 54% (*n* = 185) including other performing arts such as dance, acting, circus arts/physical theater, magic, and musical theater. The mean age was 44.41 years (*SD* = 13.85 years, range = 18–86 years), 64% (*n* = 217) of respondents were women, and 93% (*n* = 317) identified as white. Fifty percent of respondents (*n* = 171, 50%) had advanced qualifications, and 43% (*n* = 147) had tertiary or higher qualifications. Eighteen percent (*n* = 60, 18%) lived alone, 41% (*n* = 139) lived with a partner, 19% (*n* = 66) lived with a partner plus children, 11% (*n* = 38) lived with friends, and the remainder lived with other family, in other arrangements, or did not disclose.

#### Procedure and Method

Data were collected via the *HEartS Professional* survey as introduced in Study 1. In Study 2, the focus was on the individual work and wellbeing experiences of performing arts professionals in their own words, in order to identify the subjective effects of lockdown. We therefore report on area 5 of the survey that asked two open questions: (1) ‘How has the current public health situation impacted on your arts and cultural work? We are interested in any challenges and/or opportunities that you may be experiencing in relation to your work’ and (2) ‘How has the current public health situation impacted on your health and wellbeing? We are interested in any challenges and/or opportunities that you may be experiencing in relation to your health and wellbeing’. The questions required free-text responses, with no character limit.

#### Analysis

Data from the two open questions were analyzed using an inductive thematic approach in four stages. First, all data were coded using an inductive approach in Microsoft Excel. Data from the first question (impact on work) were coded first, with code(s) generated in a bottom-up manner for each free-text response. This stage was conducted by two researchers, with one researcher conducting the initial coding for half of the responses and cross-checking the remaining half, and vice versa. Data from the second question (impact on wellbeing) were coded second and cross-checked in the same way. A total of 1,576 codes were identified by the end of this phase. Second, codes from the first question were grouped into sub-themes and overarching themes agreed between the two coders. Third, codes from the second question were grouped into sub-themes, either pre-existing from stage 2 or newly developed at this stage. Fourth, sub-themes were reviewed and a final set of five overarching themes, supported by 20 sub-themes, was developed and agreed between the two researchers.

### Results

The five overarching themes are summarized in Table 1. We provide a summary of each sub-theme below, with further indicative quotes included in Supplementary Table 7. When attributing quotes to respondents, the precise nature of their work (e.g., musician, actor, and magician) is presented where available, along with the respondents’ identified gender and their age.

**TABLE 1:** Overarching themes and sub-themes, organized according to identified challenges and opportunities.

Overarching theme	Sub-themes	Instances *n*
**Challenges (*n* = 1,257)**		
(1) Lost or uncertain work and income	(1.1) Canceled, reduced, or changed work	237
	(1.2) Uncertainty for future work	172
	(1.3) Financial concerns	130
(2) Constraints of lockdown working	(2.1) Challenges working or being at home	71
	(2.2) Struggles of online work	69
	(2.3) Difficulties maintaining skills or practices	45
	(2.4) Caring responsibilities	44
(3) Loss, threat, and vulnerability	(3.1) Reduced social connections	73
	(3.2) Lack of support and vulnerability	43
	(3.3) Sense of loss or grief	35
	(3.4) Concern for beneficiaries/loved ones	32
(4) Detrimental effects on health and wellbeing	(4.1) Anxiety	100
	(4.2) Low or unstable mood	87
	(4.3) Poorer physical health	69
	(4.4) Lack of motivation or focus	50
**Opportunities (*n* = 319)**		
(5) Professional and personal opportunities	(5.1) Coping fine or living more healthily	103
	(5.2) More time and less pressure	92
	(5.3) New possibilities and activities	55
	(5.4) Enhanced social connections	44
	(5.5) New skills	25

*n refers to instances and not to participants, as some participants contributed multiple codes to a sub-theme.*

#### Theme 1: Lost or Uncertain Work and Income

The most frequently cited challenge was a loss of work (sub-theme 1.1). This manifested largely in canceled or reduced work, particularly for performing activities:

My partner and I have both lost ALL our work for the foreseeable future as professional magicians. [Magician, female, age 47]

I’ve slowly been building up work to earn a decent wage. Not only has work been canceled that I had booked in until July but this will affect my work for the foreseeable future, long after the health situation is in control. [Actor, male, age 34]

Also included in this sub-theme was reference to rapidly changed work in response to the health crisis:

Most of my work is teaching in relation to theater/performance in HE [higher education]. My teaching has been radically affected by the crisis. I was making performance with students; their assessment, teaching, and other practices have changed almost entirely. I have had to devote a lot of additional time to student support. [Theater and live arts educator/researcher, female, age 52]

Linked with the above, the second most frequently cited challenge was uncertainty regarding future work (sub-theme 1.2). This included concerns around when and how employment may return, as well as the precariousness of this for freelancers:

Not sure how the industry will sustain itself or how quickly it will be able to recover once we get through this. [Actor, male, age 44]

My performing career as a freelancer has completely stopped, and there is no indication of when this might begin again. When it does pick up, there is no guarantee that I will be booked for anything. [Musician, female, age 35]

For some, the uncertainly also extends to the future of the sector more widely as well as related worries regarding career progression:

I feel… suspended. It feels like our profession as performing artists doesn’t exist for the time being. And it feels it will take long and slow to restart. [Performing arts professional unspecified, female, age 45]

Thinking of a different occupation from the one I have devoted my life to since [childhood] is the most excruciating thing I have ever had to do. [Musician, female, age 39]

Closely linked with loss of work and uncertainty are concerns about loss of income (sub-theme 1.3):

It’s been a disaster. An utter disaster. My industry disappeared overnight. Myself and my husband had two projects lined up (he had literally just started rehearsing his) all gone. All my freelance work gone. This will set us back at least £50k [thousand]. [Performing arts professional unspecified, female, age 44]

I don’t think the current financial assistance properly takes into account those in my position who work as a freelance musician and who are also employed. My freelance earnings for the summer as a performing musician (and realistically probably well beyond) have been decimated (a £15k [thousand] loss to date − at a time which is usually the busiest in the year) and income from a regular teaching post is only paid over 10 months. I will therefore receive hardly any income in August and September but do not qualify for any help. [Musician, male, age 50]

These two examples draw attention to the rapid change in financial circumstances for some performing arts professionals, as well as the particularly precarious situation for freelancers.

#### Theme 2: Constraints of Lockdown Working

Alongside changing work profiles and the associated uncertainty and financial loss, respondents also identified challenges of working or being at home (sub-theme 2.1). These included boredom, fatigue, increased workload, and difficulties adjusting to the new circumstances:

[I am] so busy. I am trying to homeschool, keep fit, run the household, source food (so much more time consuming now), keep in touch with isolated relatives and friends as well as making sure my work does not suffer. I feel I do not have enough hours in the day. [Musician, female, age 44]

Having to work from home (where I don’t yet have a proper set up) has been a big struggle again to get motivated, or to feel like I’m not letting people down by not doing my work. There has been a lot of admin to sort out in the last few weeks, and it has been hard to prioritize at the same time as looking after the only other member of my household (family) who was quite ill. Not knowing if the symptoms for myself and family are COVID-19 or something else was mentally difficult. It’s also really difficult being restricted to one outing a day – I haven’t managed to even get out for a walk around the block in the last 5 days as I’ve felt too rubbish. [Arts administrator, female, age 30]

Furthermore, respondents identified specific challenges connected with needing to work online (sub-theme 2.2):

All of my work has moved online, and since I work mostly in a teaching capacity, I have struggled with changing lectures and workshops into this format and, as a freelancer lecturer, cannot access training for these things. [Producer, male, age 28]

Respondents also commented that, in the performing arts, online interactions are no substitute for face-to-face:

We are now doing a very small portion of our work online but it is not the same as doing it live. It’s the difference between having a conversation with a stranger and recording a video for a stranger. [Actor, gender non-identified, age 49]

Not having an audience is a huge challenge so the weekly community concert has kept us sane. [Community music practitioner, female, age 47]

Alongside challenges of being at home and working online, respondents cited the loss of opportunities to continue their professional activities. This was the case for activities conducted with others such as rehearsal but also for activities which can be individual such as practice, research, or study (sub-theme 2.3):

I am unable to sing in choirs. I am unable to practice the organ, as the instrument I use is in a church, and all churches are currently closed. I am unable to research undigitised primary resources held in libraries that are currently shut. [Musician, male, age 38]

I cannot practice anymore (my dance style can’t be practiced in my home as it’s too loud and needs too much space). [Dancer, female, age 32]

For some, this was reported as a feeling of stagnation, or it stifled creativity:

[It’s a] really bleak period: hard to be creative. [Musician, male, age 22]

Because of my age I am forced to self-isolate. I am alone in my flat and although I am very busy with online streaming with friends and other comedians, I am hungry for human contact. One begins to forget that there are others out there and that the world is not just you alone. I am a writer and a communicator and I feel stifled. [Burlesque/cabaret performer, female, age 86]

Finally, a group of participants reported challenges associated with their caring responsibilities, typically for children or other members of their family (sub-theme 2.4):

Trying to work from home, homeschool and look after 2 children and look after my own fitness and mental wellbeing have been my biggest challenges. [Performing arts professional unspecified, female, age 39]

Our trio will meet soon for rehearsals of our August and September programs in the garden (keyboard with extension lead) which we are looking forward to. On the positive side, we may be able to update or add to our tech skills by recording for radio transmission and videoing some of our performances. However, all three of us, being female, have found we have less time for practice whilst looking after our families. Without regular (5+ per month) performances, we are possibly becoming de-skilled. [Musician, female, age 59]

Of the 44 pieces of data coded to this sub-theme, 36 are attributed to women.

#### Theme 3: Loss, Threat, and Vulnerability

Respondents reported several areas in which they experienced loss, including through reduced or limited social connections (sub-theme 3.1):

A freelance musician is by their very nature a sociable and well connected person, and so many are struggling with the sheer contrast of going from seeing other like-minded individuals everyday to not seeing them at all for a considerable amount of time. [Musician and arts administrator, male, age 20]

In addition to the loss of social networks, some respondents also cited the loss of shared in-person creative experiences:

The biggest impact is for my choir. The well documented benefits of singing in a choir are so important in my life in normal times that, while I am running online sessions and they are fun, up to a point, they can’t have the amazing buzz and social interaction of proper rehearsals. [Musician, female, age 53]

A second sub-theme in this category was a perceived lack of support and feelings of vulnerability and threat (sub-theme 3.2). This manifested first through a sense that the already precarious nature of employment in the performing arts, including working unpaid in order to generate future work, was exacerbated by the pandemic:

Working freelance in the performing arts is always a precarious business. You are constantly trying to make ‘things’ happen with very little financial gain. You write, you attend auditions and workshops, you establish theater companies and apply for funding… none of these activities are paid. You might get a piece of writing acknowledged in a competition, you might get the part, you might be awarded some money. You put out in the community to provide opportunities for others… you provide invisible earnings. Now it is just harder. Almost impossible. [Actor, female, age 62]

Playing work is zero and is on rolling cancelation as the lockdown continues. Freelance orchestral work is now being offered on a ‘pencil it in’ basis so that if the lockdown isn’t over by then the work can be rubbed out by the orchestras with no question of cancelation fees – understandable, they’re potentially in financial trouble too. From choice my teaching has been in a state school with students whose parents have little money at the best of times. Lessons were heavily if not totally subsidized by the school so even pre-crisis these parents were not in a position to pay for online lessons. I am therefore now doing a small amount of free online teaching to keep them interested and motivated (I hope!). [Musician, female, age 59]

Furthermore, there was also a perceived lack of communication or support (at that time) from the government, and a feeling of being undervalued or ‘falling through the cracks’:

It makes me question the point of being in the arts when there is little to no governmental or even general public support. [Musician, female, age 22]

Having contracted COVID-19 I have certainly been fearful and anxious. I felt like I was walking into a death trap working in a care home as the PPE [personal protective equipment] is not in wide use and getting COVID-19 seems to be ‘it may or may not be serious’ scenario. I have only received SSP [statutory sick pay] while I have been off work. I have savings so this hasn’t massively worried me but does feel a bit of a kick in the teeth after putting myself and family at risk by going to work. [Music therapist, male, age 34]

Linked with this is a sense of loss, connected with self-worth and identity as well as missing professional and personal activities (sub-theme 3.3):

How to have a sense of self-worth when your job does not exist anymore? [Writer, TV, and radio presenter, female, age 41]

I am also in a kind of mourning for the industry which I know will never be the same. And there is a huge sense of grief and foreboding for all of my peers and the many wonderful organizations that I work with. [Arts administrator, female, age 32]

Finally, some respondents also expressed concern and uncertainty about the health of others, most notably loved ones or those with whom they would normally work (sub-theme 3.4):

I run a music project with vulnerable/excluded young people. We have had to ‘pause’ the project for 3 months. It affects my income, but more so, it upsets me that we cannot deliver a meaningful project to young people who need it. [Community music practitioner, female, age 53]

[I am] very afraid and worried for my elderly mother [for] whom I am primary carer. Having to also manage and deal with the stress and distress of my partner (also in the arts and out of work = also no income!) has added to my own. [Performing arts professional unspecified, male, age 42]

What we see in these sub-themes is a very tangible sense of threat, both in terms of respondents feeling isolated, unsupported, and in a precarious position but also in terms of losing key aspects of identity as a result of such a rapidly changed and reduced professional context which also comes with additional concerns for others.

#### Theme 4: Detrimental Effects on Health and Wellbeing

The effects of the lockdown – as described in the above themes – had negative implications for health and wellbeing, including anxiety (sub-theme 4.1):

I’ve had increased anxiety and that is not something I commonly suffer from. I feel very stressed not only about the future of my career, but also my health, my finances, and the wellbeing of my family and friends. [Musician, female, age 26]

Low health, feeling trapped, a lot of anxiety. [Musician, non-binary, age 21]

In addition to anxiety, respondents described low mood, worsening or new symptoms of depression, and unstable or fluctuating moods (sub-theme 4.2):

I am mentally breaking down every day. I am trying to care for my 6 year old and keep my business afloat with no income on an open ended time span. I am depressed and struggling every moment of every day. It is horrendous and has destroyed my life. [Arts administrator, female, age 46]

I have had spells of depression since the beginning of the pandemic as I have been forced to face the prospect that I will be unable to do the job I love and have trained for in the future. [Musician, female, age 28]

Alongside psychological effects, respondents reported worsening physical health including poor sleep and lack of exercise (sub-theme 4.3):

The lack of exercise in a pool is starting to impact on how I feel both physically and mentally. My sleep has been affected. I no longer fall asleep easily, and I have a few times woken up from troubling dreams related to the current situation. [Musician, female, age 40]

Eating more than usual. Drinking more than usual. Putting on some weight. Unable to be outdoors as much as usual. Not able to take part in a weekly group Tai Chi class. [Actor, male, age 59]

Finally, the impacts on health and wellbeing also extend to difficulties concentrating and staying motivated (sub-theme 4.4):

The real challenge is keeping yourself motivated/occupied. With all work being canceled for the foreseeable future, it’s difficult some days to know what to do with yourself. [Musician and researcher, male, age 27]

I feel moody, anxious, I can hardly push myself to do something productive. Everything seems pointless and I feel forgotten. [Musician and dancer, female, age 22]

#### Theme 5: Professional and Personal Opportunities

Although not as frequently mentioned as the challenges, respondents identified 319 instances of opportunities afforded by the COVID-19 lockdown. This included reporting that the situation had no impact in terms of wellbeing (“I am OK thanks” [Musician, male, age 50]) or reporting that it had provided opportunities to live a healthier lifestyle (sub-theme 5.1):

I am healthy and have more time to rest and do sports, so I am probably a bit healthier than before. No traveling and good night sleeps help as well. I feel fine! [Musician, female, age 34]

Health is fine. I am getting more exercise than before. [Musician, male, age 61]

Additionally, some respondents commented on feeling that they have more time to themselves, as well as less pressure on their day-to-day and professional lives (sub-theme 5.2):

I have worked non-stop since 16 [years old], and this has made me step back and take a break to spend time with my family. It’s a horrible situation and it’s scary to think what could happen but I’m not letting the worry consume my life. I am lucky to not have to worry about taking a break from work, not having any earnings for the short term. [Performing arts professional unspecified, female, age 43]

Two of our children are at home with us, and there is more time to spend with them and on activities that have long been neglected. [Musician, male, age 53]

Both of the examples here highlight the importance of context in terms of determining the impact of the pandemic, with the first respondent describing herself as ‘lucky’ not to have to worry about lost earnings, and the second respondent referring to positive social connections via his two children. However, it is important to note that this sub-theme was also evident in respondents who identified other negative impacts of the pandemic, such as lost earnings and work.

Linked with this, some respondents felt that they could use the time to explore new possibilities and activities (sub-theme 5.3), including finding new ways of working online, re-evaluating their life circumstances, and finding new creative opportunities:

I usually spend a great deal of time in the future. Researching, preparing, scheduling rehearsals, nurturing, and collaborating. There is no surity [*sic*] of anything in the future. Even agreed projects 3 years away will shunt or disappear. I have gone in to myself to re-evaluate everything. [Musician, male, age 58]

I am using the time to research as an artist my skills and abilities. I am developing new projects and paths of research. [Arts administrator, performer and educator, female, age 30]

I am actually caring quite well for my wellbeing, doing other hobbies. An opportunity from this chaos is being able to be taught classes by amazing teachers from all over the world, albeit through a screen. [Dancer, gender not disclosed, age 21]

A further area of opportunity for some respondents was enhanced social connections (sub-theme 5.4), through connections with family, other performing artists, and the local and wider communities:

It has made me feel better connected with the wider industry - I’ve had to do more online searching and have come across great resources and networks as a result. Working in the arts feels incredibly collaborative right now. My work is currently the area of my life in which I feel less anxious, most supported and the least alone. [Arts administrator, female, age 24]

My arts and cultural work has moved mainly extremely local, i.e., a horseshoe of three streets for whom we perform weekly concerts outdoors, socially distanced. The local community is much stronger for it – everyone attends regularly, there is much appreciation of the occasion to see others, the appreciation of the music is huge even though many who turn up are unlikely to have ever entered a concert hall before. [Community music practitioner, female, age 47]

Finally, a smaller group of respondents mentioned the new skills that they had acquired during the lockdown, mainly focusing on digital and creative skills (sub-theme 5.5):

Opportunities: finding new ways of doing things, applying creativity and new technological solutions [Arts administrator, male, age 30]

It has forced me to use new media and overcome challenges, learn a lot of new skills and adapt. I needed to develop a lot of patience. [Music therapist, female, age 51]

### Discussion

These qualitative analyses have revealed the subjective effects of COVID-19 Lockdown 1.0 on the work and wellbeing experiences of performing arts professionals in the UK, demonstrating that respondents identify a complex array of challenges and opportunities. Overall, the number of challenges cited far outweighed the number of opportunities, although for some respondents the effects have been a mixture of both challenge and opportunity.

Many of the challenges identified mirror those found in literature with the wider population. For example, Evanoff et al. (2020) reported that poorer mental health outcomes during the pandemic were associated with home or family stressors. Theme 2 highlights some of the home-specific stressors faced by respondents in this study, including caring responsibilities for children and other relatives. As noted in the results, the majority of respondents contributing to this sub-theme were women. While this may be a function of the bias toward women in the sample, it may also reflect a gendered impact of the pandemic when it comes to caring responsibilities. For example, using panel data from the United States Current Population Survey, Collins et al. (2020) report that the gender gap in work hours has grown by 20–50 percent, with mothers of young children reducing their working hours four to five times more than fathers. Further research will be required in order to probe gender-related impacts of COVID-19 in the performing arts. Other negative psychological implications of quarantine identified by Brooks et al. (2020), such as boredom, poor information, and financial loss, are also seen in this current sample, as is evidence of anxiety and depressive symptoms reported by Fancourt et al. (2021).

However, some of the challenges and opportunities identified in this study appear more specific to the nature of the work in which respondents are engaged. Theme 1, for example, captures respondents’ experiences of lost work that speak very directly to the precarious working conditions and working structures of many in the performing arts. This resulted, as shown, in some respondents literally losing all of their booked work overnight, without the structure of a contract or salary to provide even short-term security, and a feeling among some respondents of being ‘forgotten’ or unsupported. Self-employed workers, workers not paid a salary, workers with variable hours, and those unable to work from home are more likely to have been negatively affected by the economic downturn (Adams-Prassl et al., 2020). This is of relevance here given that artists (including performing artists) were identified by Woronkowicz and Noonan (2017) as being disproportionately freelance, and more likely to move in and out of self-employment, when compared with other professional workers. This structural precariousness in the performing arts sector is brought to light by the pandemic but it is not new, a point made by Comunian and England (2020) in their argument that “in order to value and expect resilience, there is a need to reflect on how – before any shock – the [creative and cultural work] system is supported and developed within a sustainable framework” (p. 123).

The loss of work also had implications for the maintenance of skills (sub-theme 2.3), particularly for those who require resources such as venues or instruments that were inaccessible during lockdown. For those who would normally perform in a venue (e.g., a theater), the lockdown effectively prevented their artistic practice entirely. Sub-theme 4.4 captured how this also intersected with motivation, with some respondents reporting that it was difficult to practice or to work when there was little promise of any immediate or even medium-term prospects for their pre-COVID-19 practices to resume. While many respondents adapted where they could, for example through teaching or performing online, this was not possible for everyone. When it was possible, it often required rapid skill development and/or felt like a poor substitute for face-to-face activities. Indeed, the lack of audience was specifically drawn out as a point in sub-theme 2.2, and the lack of, or reduced, social connections in sub-theme 3.1. This is particularly concerning given that previous research has indicated that shared creative moments are important in sustaining wellbeing for musicians (Ascenso et al., 2017, 2018), with the respondents to this survey indicating that such moments were harder to access during lockdown.

A further aspect of note is the profound feeling of loss and threat reported by the respondents. Sub-theme 1.2 indicates not only uncertainty regarding individual future employment but also uncertainty regarding the very future of the arts and cultural sector. Sub-theme 3.3 illustrates how these sorts of meta-uncertainties also sit alongside very personal experiences of what respondents term ‘grief’ at the losses that the lockdown has imposed on those working in the performing arts. This reminds us of how closely intertwined wellbeing and identity are with performing arts professionals’ work activities (see also Teague and Smith, 2015), a point also highlighted by the lack of motivation discussed under sub-theme 4.4. The inability to work appears to be, for many in this sample, detrimental not only to their wellbeing and financial situation but also to their very sense of self and their motivation.

It is important to recall, however, that the respondents also identified opportunities associated with lockdown and were resilient in finding solutions to its immediate challenges. For some, lockdown provided a period of relative calm in what was previously a busy and stressful working life, allowing for more ‘me time.’ Returning to the discussion above regarding the nature of careers in the performing arts, this reinforces the point that structural aspects of working lives – such as overwork – were present pre-COVID-19. The ways in which people were able to respond will also be different depending on individual circumstances. For example, while the respondents cited in sub-theme 5.2 pointed to elements of ‘luck’ or circumstances that they could positively exploit during lockdown, this will not be the case for many others. There is already concern that the pandemic will reinforce inequalities in the cultural sector: “without policy intervention, it is likely that disproportionately more workers from already under-represented groups, and especially those who belong to more than one under-represented group, will drop out of the cultural economy workforce” (Eikhof, 2020, p. 239). Further research exploring the ongoing impact of the pandemic on issues of equality within the cultural workforce will be needed, alongside specific studies aimed at understanding why and how some performing artists have adapted to the circumstances more readily than others. Shanahan et al. (2020), for example, suggest that daily routine, physical activity, and positive reframing are coping strategies used by young people during the pandemic associated with reduced distress. Further work will help to explain how such strategies, as well as others seen in this study such as focusing on new skill development or new activities, could extend more widely to those working in the performing arts and what barriers might prevent their implementation.

Study 2 provides insight into the impacts of Lockdown 1.0 as they relate to the multi-faceted and often freelance nature of performing arts professionals’ working lives, their reliance on face-to-face collaborations and interactions, and the close links between their profession and their identity and wellbeing. The new-found ‘me time’ and ability to prioritize healthier lifestyles is also of note, providing insight into how future professional lives can be optimized. This fits well with the finding by Willis et al. (2019) that music-making, performance activities, and social support were identified by performing artists as important for better wellbeing, and finding new ways to enable and support these, while also reinstating job security and income, will be important for the sector moving forward. Further research should focus on a wider demographic and take a longitudinal approach in order to understand how these initial effects change, or not, as the course of the pandemic continues.

## General Discussion

The findings from the two studies presented here illustrate the range of ways in which COVID-19 Lockdown 1.0 impacted on performing arts professionals. While a large number of challenges were identified, the respondents also pointed out some areas of opportunity and were, to some extent, resilient in their adaptation. Among the challenges, many – such as canceled work, unexpected loss of income, challenges of working from home, and feelings of loss and grief – were linked to the immediate effects of the pandemic. Others – such as the precariousness of freelance working and mental health problems – were also challenges pre-COVID (Bartleet et al., 2019; Willis et al., 2019) and appeared to be exacerbated by the impact of the lockdown. As the pandemic progresses, performing arts professionals continue to face closed venues, canceled work, and the restrictions of social distancing. Without financial and structural support, tailored to the nature of performing arts professionals’ often freelance or self-employed profiles, many may not be able to stay in the profession and many venues may be forced to close.

There is, of course, much variety within the arts sector and many professionals’ situations are specific to them or a subgroup of professionals like them. This means that blanket solutions will be difficult to find: for example, some respondents’ work transferred online relatively easily while others’ work did not, and some respondents reported caring responsibilities or living situations that presented additional challenges to their pandemic response. Key to how the sector responds to the pandemic is a recognition, first, that structural insecurities in the performing arts sector are not new (e.g., Comunian and England, 2020) and, second, that different individuals will be working in different professional and personal contexts, all of which are influenced by wider issues of inequality (e.g., Eikhof, 2020). Nonetheless, the evidence presented here points to a number of implications for those working in the sector.

First, the results from both studies point to significant wellbeing concerns within the respondents, relating to financial, social, and mental health. This highlights the importance for those who educate and work with performing artists to engage in cross-discipline initiatives such as *Healthy Conservatoires*^1^, a network which supports environments that promote and enhance the health and wellbeing of performing artists, enabling them to achieve their full potential and to build healthy and sustainable careers. Sector-specific networks such as this are well placed to identify ongoing and specific challenges faced by performing arts professionals such as grief, lack of motivation, and loss of identity, to share best practices in responding to these challenges and adapting to the pandemic, and to lobby as needed for government support packages that meet these professionals’ needs. Second, despite the majority of respondents reporting increased anxiety, as well as low and unpredictable mood, over half did not seek help for health and wellbeing. This suggests that respondents may have been employing their own coping strategies or that they did not know about available support, did not wish to access it, or found that there were barriers to accessing it. This may have changed since these data were collected and subsequent research should uncover how performing arts professionals are managing their health and wellbeing as the pandemic continues, their coping strategies, and any barriers they experience in accessing support. Finally, the associations between physical activity and wellbeing identified in Study 1 are in line with previous research that suggests the importance of physical health and fitness for performing artists (Cahalan and O’Sullivan, 2013; Donohue et al., 2018; Araújo et al., 2020) and in the general population during COVID-19 (Wood et al., 2020). The opportunity to exercise more was identified as one of the opportunities afforded by lockdown in Study 2, though for other respondents exercise routines were prevented by lockdown and may continue to be affected by ongoing restrictions. We would suggest that performing arts professionals consider integrating physical activity into their daily routines where possible, both in order to achieve targeted performance-related fitness and better health in general but also with the aim of maximizing wellbeing and social connectedness, as well as minimizing loneliness and depression, during times of crisis.

This research is limited by its cross-sectional design, and our convenience sample was relatively highly educated, had a high proportion respondents who were white, female, from London, with relatively high incomes and included a relatively large proportion of classical musicians. As ethnic minorities have been disproportionately impacted by COVID-19 in the UK (Platt and Warwick, 2020), further investigation is needed to reveal the specific challenges that performing arts professionals within these groups may face. Our experiences in tackling our original recruitment aims – to reach a broad range of performing arts professionals from a variety of backgrounds – may speak to some of the challenges facing this group of often freelance practitioners who are not necessarily connected to large organizations, with many more suddenly furloughed or unemployed. A follow-up study with a broader range of respondents is urgently needed.

Nonetheless, these findings provide a snapshot of the experience of a group of people working in the performing arts at the start of the COVID-19 pandemic in the UK. The rapid reductions that we saw in mental and social wellbeing as lockdown took hold are concerning and reflect the unstable nature of the situation and the importance of continuing to trace the impacts on performing arts professionals as the situation develops. While the *HEartS Professional Survey* is able to capture key indicators of experience, in-depth discussion with performing arts professionals will also be essential in understanding how individuals are experiencing, perceiving, and responding to this changing situation.

## Data Availability Statement

The dataset generated for Study 1 can be found in an online repository. The names of the repository and accession number can be found below: https://doi.org/10.5061/dryad.s7h44j14z.

## Ethics Statement

The studies involving human participants were reviewed and approved by the Conservatoires UK Research Ethics Committee. The participants provided their written informed consent to participate in this study.

## Author Contributions

NS, RP, UT, AM-B, IC, SG, and AW: conceptualization, methods and data collection, and comments on manuscript. NS, SK, and RP: data curation and data analysis. NS, RP, and AW: manuscript first draft. AW, RP, and NS: secured funding. All authors contributed to the article and approved the submitted version.

## Conflict of Interest

The authors declare that the research was conducted in the absence of any commercial or financial relationships that could be construed as a potential conflict of interest.

## Footnotes

1 www.healthyconservatoires.org
