# Supplementary material for: The Effects of COVID-19 Lockdown 1.0 on Working Patterns, Income, and Wellbeing Among Performing Arts Professionals in the United Kingdom (April–June 2020)
Source: Front Psychol. 2021 Feb 10;11:594086. doi: 10.3389/fpsyg.2020.594086 (PMC7902701; doi:10.3389/fpsyg.2020.594086)
Supplement: Supplementary file 1 [file Table_1.pdf]

Spiro N, Perkins R, Kaye S, Tymoszuk U, Mason-Bertrand A, Cossette I, Glasser S, and Williamon A (2021), The Effects of COVID-19 Lockdown 1.0 on Working Patterns, Income, and Wellbeing among Performing Arts Professionals in the United Kingdom (April–June 2020), *Front. Psychol.* 11:594086. doi: 10.3389/fpsyg.2020.594086.

SUPPLEMENTARY TABLE 1 | Sociodemographic, economic characteristics, and experience of COVID-19 of the sample, *HEartS Professional Survey*, *N*=385.

	n	%
**When was this survey completed?**		
Block 1 (1–15 April 2020)	189	49%
Block 2 (16–30 April 2020)	123	32%
Block 3 (1–15 May 2020)	21	6%
Block 4 (16–31 May 2020)	16	4%
Block 5 (1–15 June 2020)	36	9%
**Region (see Supplementary Figure 1, *HEartS Professional Survey*, question 2.1)**		
Northern Scotland	1	0.3%
Southern Scotland	26	7%
North East	5	1%
North West	15	4%
Yorkshire and the Humber	14	4%
East Midlands	12	3%
West Midlands	16	4%
East of England	28	7%
South East	62	16%
South West	28	7%
London	162	42%
North Wales	2	1%
South Wales	12	3%
Northern Ireland	2	1%
	**Mean**	***SD***
**Age (see Supplementary Figure 1, *HEartS Professional Survey*, question 2.2)**	44.08	13.9
**Categories**	***n***	**%**
18-25	34	9%
26-35	90	23%
36-45	89	23%
46-55	89	23%
56-65	57	15%
66-75	23	6%
76-94	3	1%
**Gender (see Supplementary Figure 1, *HEartS Professional Survey*, question 2.3)**		
Men	135	35%
Women	242	63%
Non-binary/transgender	7	2%
Prefer not to say	1	0.3%

	n	%
**Ethnicity (see Supplementary Figure 1, *HEartS Professional Survey*, question 2.4)**		
White British or Irish	303	79%
Any other White background	54	14%
Mixed ethnic backgrounds*	17	4%
Asian ethnic backgrounds*	2	1%
Black ethnic backgrounds*	2	1%
Any other ethnic background	7	2%
**Professional specialisms (see Supplementary Figure 1, *HEartS Professional Survey*, question 2.5 and 4.1)**		
**Music or sound arts**	**260**	**68%**
Classical	155	60%
Dance (including electronic, house, techno)	2	1%
Jazz / blues / R'n'B	1	0.4%
Pop / rock	1	0.4%
Church / gospel	8	3%
Music therapy / community music	11	4%
Film / TV / theatre / radio / video games	9	4%
Other	16	6%
Not specified	57	22%
**Performing arts**	**201**	**52%**
Acting	33	16%
Dancing	10	5%
Circus arts / physical theatre	3	2%
Magic	2	1%
Musical theatre	22	11%
Other	58	29%
Not specified	73	36%
**Visual arts, Crafts, and Decorative arts**	**15**	**4%**
Drawing / illustration	2	13%
Ceramics	2	13%
Design	3	20%
Textiles	1	7%
Furniture making / wood working	3	20%
Painting / photography / printmaking	5	33%
Film / video making	3	20%
Other	11	73%
**Literature**	**13**	**3%**
Journalism	1	8%
Non-fiction	1	8%
Other	5	39%
Not specified	6	46%

	n	%
**Education (see Supplementary Figure 1, *HEartS Professional Survey*, question 2.8)**		
Secondary qualification (e.g. high school diploma)	25	7%
Tertiary / higher/ further qualification (e.g. bachelors degree)	165	43%
Advanced qualification (e.g. masters, PhD, DMA, DMus degree)	195	51%
**Living status (see Supplementary Figure 1, *HEartS Professional Survey*, question 2.9)**		
On my own	67	17%
Residential care setting	0	0%
My spouse or partner	240	62%
Children	91	27%
Other family	19	5%
Friends or house share	48	13%
Other	2	1%
Would rather not say	2	1%
**Household income (see Supplementary Figure 1, *HEartS Professional Survey*, question 5.2)**		
Up to £5,199	6	2%
£5,200 and up to £10,399	22	6%
£10,400 and up to £15,599	16	4%
£15,600 and up to £20,799	21	6%
£20,800 and up to £25,999	27	7%
£26,000 and up to £31,199	38	10%
£31,200 and up to £36,399	27	7%
£36,400 and up to £41,599	14	4%
£41,600 and up to £46,799	20	5%
£46,800 and up to £51,999	26	7%
£52,000 and up to £75,999	68	18%
£76,000 and above	70	18%
Would rather not say	30	8%
	**Mean**	***SD***
**Percentage of household income contribution (*n*=381)**	58.54	31.8
**Percentage of contribution funded through arts work (*n*=382)**	76.47	33.8
**COVID-19 (see Supplementary Figure 1, *HEartS Professional Survey*, question 3.1)**	**n**	**%**
Tested positive	2	1%
Have or previously had symptoms	55	14%
Not that know of	328	85%

* *Ethnicity:* Mixed ethnic backgrounds includes White and Black Caribbean, White and Black African, White and Asian, and any other mixed ethnic background; Asian ethnic backgrounds includes Indian, Pakistani, Bangladeshi, Chinese, and any other Asian ethnic background; Black ethnic backgrounds includes Caribbean, African, and any other Black ethnic background.
