# Supplementary material for: The Effects of COVID-19 Lockdown 1.0 on Working Patterns, Income, and Wellbeing Among Performing Arts Professionals in the United Kingdom (April–June 2020)
Source: Front Psychol. 2021 Feb 10;11:594086. doi: 10.3389/fpsyg.2020.594086 (PMC7902701; doi:10.3389/fpsyg.2020.594086)
Supplement: Supplementary file 2 [file Table_2.pdf]

Spiro N, Perkins R, Kaye S, Tymoszuk U, Mason-Bertrand A, Cossette I, Glasser S, and Williamon A (2021), The Effects of COVID-19 Lockdown 1.0 on Working Patterns, Income, and Wellbeing among Performing Arts Professionals in the United Kingdom (April–June 2020), *Front. Psychol.* 11:594086. doi: 10.3389/fpsyg.2020.594086.

SUPPLEMENTARY TABLE 2 | General health and physical fitness characteristics, *HEartS Professional Survey*, *N*=385.

									n	%
**Self-rated health[1] (see Supplementary Figure 1, *HEartS Professional Survey*, question 7.3)**										
Very good									138	39%
Good									192	50%
Fair									50	13%
Bad									4	1%
Very bad									0	0%
Would rather not say									1	0%
**Ongoing (chronic) health issues (see Supplementary Figure 1, *HEartS Professional Survey*, question 7.4)**										
No									263	68%
Yes									108	28%
Would rather not say									14	4%
Mental health problems									28	26%
Chronic musculoskeletal problems									20	19%
Cancer									1	0%
Cardiovascular disease									6	6%
Chronic respiratory disease									17	16%
Chronic pain									18	17%
Other									44	41%
More than one health condition									22	20%
**Physical activity: Frequency[2] pre-COVID 19 (see Supplementary Figure 1, *HEartS Professional Survey*, question 7.5)**										
		**Mild**		**Moderate**		**Vigorous**			**Total**	
Hardly ever or never		9	2.3%	48	12.5%	145	37.7%		202	18%
About once to 3 times a month		16	4.2%	53	13.8%	58	15.1%		127	11%
Once or twice a week		68	17.7%	127	33.0%	88	22.9%		283	25%
3 times a week or more		292	75.8%	157	40.8%	94	24.4%		583	47%
**Physical activity: Frequency post-COVID 19 (see Supplementary Figure 1, *HEartS Professional Survey*, question 7.6)**										
Much less often									88	23%
Quite a lot less often									66	17%
A little less often									40	10%
No change									50	13%
A little more often									68	18%
Quite a lot more often									45	12%
Much more often									28	7%

	n	%
**Physical activity: Medium (see Supplementary Figure 1, *HEartS Professional Survey*, question 7.7)**		
I didn't do any sports or other energetic activities	62	16%
Online alone	51	13%
Online with others	53	14%
Offline alone	156	41%
Offline with others	63	16%

[1] For general health, we used one item from the Short Form 36 (SF-36) Health Survey (Ware and Gandek, 1998).

[2] Physical activity was measured using a scale from the Whitehall II Study, which measures frequency of engagement in activities that are mildly, moderately, and vigorously energetically taxing. Frequency is rated on a 4-point scale, from 0 *Hardly ever or never* to 3 *3 times a week or more* (Marmot and Brunner, 2005).

References

Marmot M and Brunner E (2005), Cohort Profile: The Whitehall II Study, *Int J Epidemiol* 34, doi: 10.1093/ije/dyh372

Ware JE and Gandek B (1998), Overview of the SF-36 Health Survey and the International Quality of Life Assessment (IQOLA) Project, *J Clin Epidemiol* 51, doi: 10.1016/S0895-4356(98)00081-X
