# Supplementary material for: The Effects of COVID-19 Lockdown 1.0 on Working Patterns, Income, and Wellbeing Among Performing Arts Professionals in the United Kingdom (April–June 2020)
Source: Front Psychol. 2021 Feb 10;11:594086. doi: 10.3389/fpsyg.2020.594086 (PMC7902701; doi:10.3389/fpsyg.2020.594086)
Supplement: Supplementary file 3 [file Table_3.pdf]

Spiro N, Perkins R, Kaye S, Tymoszuk U, Mason-Bertrand A, Cossette I, Glasser S, and Williamon A (2021), The Effects of COVID-19 Lockdown 1.0 on Working Patterns, Income, and Wellbeing among Performing Arts Professionals in the United Kingdom (April–June 2020), *Front. Psychol.* 11:594086. doi: 10.3389/fpsyg.2020.594086.

SUPPLEMENTARY TABLE 3 | Changes in work profiles and social meetings, *HEartS Professional Survey*, *N*=385.

3A. Time spent doing areas of work since the start of COVID-19
(see Supplementary Figure 1, *HEartS Professional Survey*, question 4.4)

	Less time		No change		More time	
	n	**%**	***n***	**%**	***n***	**%**
Performing (*n*=254)	243	96%	3	1%	8	3%
Conducting / directing / producing (*n*=89)	80	90%	3	3%	6	7%
Teaching / coaching / workshop leading / mentoring (*n*=233)	169	73%	20	9%	44	19%
Managing / promoting (*n*=79)	49	62%	15	19%	15	19%
Composing / choreographing / designing / making / writing (*n*=94)	47	50%	5	5%	42	45%
Appraising / assessing / evaluating / reviewing (*n*=86)	44	51%	22	26%	20	23%
Researching (*n*=83)	33	40%	14	17%	36	43%
Curating / editing / presenting / technical supporting (*n*=49)	26	53%	10	20%	13	27%
Other (*n*=62)	43	69%	6	10%	13	21%
**Average %**		**71%**		**9%**		**19%**

3B. Medium (online or offline) and social context of work activities
(see Supplementary Figure 1, *HEartS Professional Survey*, questions 4.5 and 4.6)

	Pre-COVID-19		Currently	
	n	**%**	***n***	**%**
**Performing (*n*=254)**				
Online alone	6	2%	78	31%
Online with others	3	1%	54	21%
Offline alone	20	8%	104	41%
Offline with others	225	89%	18	7%
**Conducting / directing / producing (*n*=89)**				
Online alone	2	2%	26	29%
Online with others	4	5%	25	28%
Offline alone	10	11%	33	37%
Offline with others	73	82%	5	6%
**Teaching / coaching / workshop leading / mentoring (*n*=233)**				
Online alone	3	1%	81	35%
Online with others	10	4%	113	49%
Offline alone	43	19%	26	11%
Offline with others	177	76%	13	6%

cont…

	Pre-COVID-19		Currently	
	n	%	*n*	%
**Managing / promoting (*n*=79)**				
Online alone	12	15%	28	35%
Online with others	27	34%	45	57%
Offline alone	4	5%	5	6%
Offline with others	36	46%	1	1%
**Composing / choreographing / designing / making / writing (*n*=94)**				
Online alone	7	7%	16	17%
Online with others	3	3%	10	11%
Offline alone	69	73%	67	71%
Offline with others	15	16%	1	1%
**Appraising / assessing / evaluating / reviewing (*n*=86)**				
Online alone	13	15%	39	45%
Online with others	7	8%	29	34%
Offline alone	21	24%	15	17%
Offline with others	45	52%	3	4%
**Researching (*n*=83)**				
Online alone	35	42%	49	59%
Online with others	7	8%	14	17%
Offline alone	33	40%	20	24%
Offline with others	8	10%	0	0%
**Curating / editing / presenting / technical supporting (*n*=49)**				
Online alone	5	10%	20	41%
Online with others	8	16%	18	37%
Offline alone	11	22%	11	22%
Offline with others	25	51%	0	0%
**Other (*n*=62)**				
Online alone	6	10%	20	32%
Online with others	11	18%	23	37%
Offline alone	9	15%	17	27%
Offline with others	36	58%	2	3%

3C. Skill maintenance and development
(see Supplementary Figure 1, *HEartS Professional Survey*, questions 4.7 and 4.8)

**Learning / practising / preparing / reflecting…**	**individually**		**with others online**		**with others in person**		**overall**	
	n	%	*n*	%	*n*	%	mean *n*	mean %
I haven't done it	37	10%	104	27%	242	61%	127.7	33%
Less*	163	42%	80	21%	116	30%	119.7	31%
No change	63	16%	31	8%	20	5%	38.0	10%
More*	122	32%	170	44%	7	2%	99.7	26%

3D. Changes in socializing
(see Supplementary Figure 1, *HEartS Professional Survey*, questions 3.4 and 3.5)

	In person		Electronically		Total (%)	
	n	**%**	***n***	**%**	***n***	**%**
Fewer people*	366	95%	21	6%	270	70%
No change	4	1%	24	6%	91	24%
More people*	15	4%	340	88%	24	6%

3E. Changes in finance
(see Supplementary Figure 1, *HEartS Professional Survey*, question 5.6)

	n	**%**
**Increase:**	**11**	**3%**
£2000+	1	0.3%
£1000 – £1999	1	0.3%
£1 – £999	9	2%
**No change:**	**82**	**21%**
**Decrease:**	**292**	**76%**
£1 – £999	60	16%
£1000 – £1999	53	14%
£2000 – £2999	45	12%
£3000 – £3999	25	7%
£4000+	109	28%

3F. Financial hardship
(see Supplementary Figure 1, *HEartS Professional Survey*, question 5.7)

	n	**%**
**Yes**	**208**	**54%**
Yes, a lot	58	15%
Yes, a little	145	38%
No	182	47%

3G. Changes in loneliness and anxiety
(see Supplementary Figure 1, *HEartS Professional Survey*, questions 3.6 and 3.7)

	Lonely		Anxious	
	n	**%**	***n***	**%**
More*	244	63%	328	85%
No change	114	30%	42	11%
Less*	27	7%	15	4%

* *For Tables 3A, 3C, and 3E:* 'More' includes 'Much more, quite a lot more, and a little more'; 'Less' includes 'Much less, quite a lot less, and a little less'. *For Table 3D:* 'Fewer people' includes 'Substantially fewer people, far fewer people, and fewer people'; 'More people' includes 'Substantially more people, far more people, and more people'.
