# Supplementary material for: The Effects of COVID-19 Lockdown 1.0 on Working Patterns, Income, and Wellbeing Among Performing Arts Professionals in the United Kingdom (April–June 2020)
Source: Front Psychol. 2021 Feb 10;11:594086. doi: 10.3389/fpsyg.2020.594086 (PMC7902701; doi:10.3389/fpsyg.2020.594086)
Supplement: Supplementary file 4 [file Table_4.pdf]

Spiro N, Perkins R, Kaye S, Tymoszuk U, Mason-Bertrand A, Cossette I, Glasser S, and Williamon A (2021), The Effects of COVID-19 Lockdown 1.0 on Working Patterns, Income, and Wellbeing among Performing Arts Professionals in the United Kingdom (April–June 2020), *Front. Psychol.* 11:594086. doi: 10.3389/fpsyg.2020.594086.

SUPPLEMENTARY TABLE 4 | Mental health, wellbeing, and social outcome measures, *HEartS Professional Survey*, *N*=385.

**Mental Health Continuum - Short Form (MHC-SF) 14-item scale[1] (see Supplementary Figure 1, *HEartS Professional Survey*, question 7.1)**		
Score (0–70): Mean, *SD*	41.12	13.32
Categorical variable: *n*, %		
Languishing	43	11%
Moderate wellbeing	210	55%
Flourishing	132	34%

**Centre for Epidemiologic Studies Depression Scale (CES-D) Short Form 8-item scale[2] (see Supplementary Figure 1, *HEartS Professional Survey*, question 7.2)**		
Score (0–8): Mean, *SD*	4.03	2.35
Number of depression cases (≥ 3 score): *n*, %	266	69%

**Social Connectedness Scale-Revised (SCS-R) 15-item scale[3] (see Supplementary Figure 1, *HEartS Professional Survey*, question 8.1)**		
Score (0–75): Mean, *SD*	48.56	12.41

**Three-Item Loneliness Scale (UCLA)[4] (see Supplementary Figure 1, HEartS Professional Survey, question 8.2)**		
Score (3-9): Mean, *SD*	5.12	1.66
Number of lonely cases (≥6 score): *n,* %	157	41%

**Spearman's rho correlation between outcome measures***	Centre for Epidemiologic Studies Depression Scale	Social Connectedness Scale-Revised	Three-Item Loneliness Scale (UCLA)
Mental Health Continuum Short Form	-.55	.59	-.49
Centre for Epidemiologic Studies Depression Scale		-.41	.48
Social Connectedness Scale-Revised			-.61

[1] Higher scores indicate better wellbeing (Keyes 2002, 2005).
[2] Scores of 3 or more indicate depression (Karim et al., 2015).
[3] Higher scores indicate better levels of social connectedness (Lee et al., 2008).
[4] Scores of 6 or higher indicate loneliness (Steptoe et al., 2013).
*$p < .001$.

References

Karim, J., Weisz, R., Bibi, Z., & ur Rehman, S. (2015), Validation of the eight-item Center for Epidemiologic Studies Depression Scale (CES-D) among older adults, *Current Psychology* 34, 681–692. doi: 10.1007/s12144-014-9281-y.

Keyes CLM (2002), The mental health continuum: From languishing to flourishing in life, *J Health Soc Behav* 43. doi: 10.2307/3090197.

Keyes CLM (2005), Mental illness and/or mental health? Investigating axioms of the complete state model of health, *J Consult Clin Psychol* 73. doi: 10.1037/0022-006X.73.3.539.

Lee RM, Dean BL, and Jung K-R (2008), Social connectedness, extraversion, and subjective well-being: Testing a mediation model, *Pers Individ Dif* 45. doi: 10.1016/j.paid.2008.05.017.

Steptoe A, Shankar A, Demakakos P, and Wardle J (2013), Social isolation, loneliness, and all-cause mortality in older men and women, *Proc Natl Acad Sc*i 110, 5797–5801. doi:10.1073/pnas.1219686110.
