# Supplementary material for: The Effects of COVID-19 Lockdown 1.0 on Working Patterns, Income, and Wellbeing Among Performing Arts Professionals in the United Kingdom (April–June 2020)
Source: Front Psychol. 2021 Feb 10;11:594086. doi: 10.3389/fpsyg.2020.594086 (PMC7902701; doi:10.3389/fpsyg.2020.594086)
Supplement: Supplementary file 5 [file Table_5.pdf]

Spiro N, Perkins R, Kaye S, Tymoszuk U, Mason-Bertrand A, Cossette I, Glasser S, and Williamon A (2021), The Effects of COVID-19 Lockdown 1.0 on Working Patterns, Income, and Wellbeing among Performing Arts Professionals in the United Kingdom (April–June 2020), *Front. Psychol.* 11:594086. doi: 10.3389/fpsyg.2020.594086.

SUPPLEMENTARY TABLE 5 | Multiple linear regression modelling four outcomes, *n* = 353.

5A. Multiple linear regression modelling wellbeing

	**Model 1**						**Model 2**					
	B	*SE B*	β	*p*	95% CI		*B*	*SE B*	β	*p*	95% CI	
(Constant)	42.98	1.57		.000	39.89	46.06	23.69	5.18		.000	13.50	33.89
COVID-19-Specific												
**Timeframe**	**3.86**	**1.37**	**0.15**	**.005**	**1.16**	**6.55**	**4.42**	**1.42**	**0.17**	**.002**	**1.63**	**7.21**
**Lockdown exercise**	**1.23**	**0.34**	**0.19**	**<.001**	**0.56**	**1.91**	**1.25**	**0.34**	**0.19**	**<.001**	**0.57**	**1.93**
Socializing change	0.11	0.41	0.01	.789	-0.69	0.91	0.36	0.41	0.05	.389	-0.46	1.16
**Financial hardship**	**-1.21**	**0.41**	**-0.15**	**.003**	**-2.01**	**-0.41**	**-1.28**	**0.46**	**-0.16**	**.006**	**-2.18**	**-0.37**
Demographics												
Gender							-0.51	1.42	0.02	.723	-2.30	3.31
Ethnicity							-0.28	2.54	-0.01	.914	-5.28	4.73
**Age**							**0.21**	**0.06**	**0.21**	**<.001**	**0.10**	**0.31**
**Health**							**2.54**	**0.99**	**0.14**	**.011**	**0.59**	**4.50**
**Pre-COVID-19 exercise**							**0.64**	**0.24**	**0.15**	**.008**	**0.17**	**1.11**
Living alone							-2.78	2.18	-0.08	.204	-7.07	1.51
Ed. attainment							0.91	1.40	0.03	.516	-1.85	3.67
Household income							0.00	0.24	0.00	.991	-0.48	0.48
% Freelance							0.02	0.02	0.06	.310	-0.02	0.06
% Cont. income							0.04	0.03	0.10	.125	-0.01	0.10
% Cont. from art							-0.01	0.02	-0.02	.661	-0.05	0.03
Arts area							-1.22	1.93	-0.03	.529	-5.01	2.58
R^2	.085						.170					
Adjusted R^2	.074						.131					
ΔR^2	.085						.085					
F	8.08***						4.32***					
ΔF							2.88***					

Abbreviations: B, unstandardized beta; SEB, standard error of B; β, standardized beta, CI, confidence interval.
Note: *N*=353, **p* < .05, ***p* < .01, ****p* < .001, Gender = male, Ethnicity = white. Starting model: df (4, 352), VIF<1.03, Tol>0.98. Final Model: *df* (16, 352), VIF <1.76, Tol>0.57. Durbin-Watson = 2.02.

5B. Multiple linear regression modelling depression

	Model 1						Model 2					
	B	*SE B*	β	*p*	95% CI		*B*	*SE B*	β	*p*	95% CI	
(Constant)	3.58	0.28		.000	3.03	4.13	6.30	0.91		.000	4.51	8.09
**COVID-19-Specific**												
Timeframe	-0.34	0.25	-0.07	.163	-0.83	0.14	-0.49	0.25	-0.10	.052	-0.99	0.01
**Lockdown exercise**	**-0.19**	**0.06**	**-0.16**	**.002**	**-0.31**	**-0.07**	**-0.16**	**0.06**	**-0.13**	**.010**	**-0.28**	**-0.04**
Socializing change	0.10	0.07	0.07	.186	-0.05	0.24	0.03	0.07	0.02	.688	-0.11	0.17
**Financial hardship**	**0.24**	**0.07**	**0.17**	**.001**	**0.10**	**0.39**	**0.18**	**0.08**	**0.13**	**.030**	**0.02**	**0.33**
**Demographics**											.	
**Gender**							**-0.69**	**0.25**	**-0.14**	**.006**	**-1.19**	**-0.20**
Ethnicity							0.53	0.45	0.06	.241	-0.35	1.41
**Age**							**-0.05**	**0.01**	**-0.29**	**<.001**	**-0.07**	**-0.03**
**Health**							**-0.41**	**0.18**	**-0.12**	**.019**	**-0.75**	**-0.07**
Pre-COVID-19 exercise							-0.03	0.04	-0.05	.414	-0.12	0.05
Living alone							0.70	0.38	0.11	.067	-0.05	1.46
Ed. attainment							-0.02	0.25	-0.00	.946	-0.50	0.47
% Freelance							0.00	0.00	0.00	.960	-0.01	0.08
Household income							0.01	0.04	0.02	.806	-0.07	0.10
% Cont. income							0.00	0.01	0.02	.735	-0.01	0.01
% Cont. from art							-0.00	0.00	-0.05	.346	-0.01	0.00
Arts area							0.23	0.34	0.04	.492	-0.43	0.90
R^2	.068						.185					
Adjusted R^2	.057						.146					
ΔR^2	.068						.117					
F	6.34***						4.76***					
ΔF							4.01***					

Abbreviations: B, unstandardized beta; SEB, standard error of B; β, standardized beta; CI, confidence interval.
Note: *N*=353, *p < .05, **p < .01, ***p < .001, Gender = male, Ethnicity = white. Starting model: df (4, 352), VIF<1.03, Tol>0.98. Final Model: *df* (16, 352), VIF <1.76, Tol>0.57. Durbin-Watson = 1.93.

5C. Multiple linear regression modelling social connectedness

	Model 1						Model 2					
	B	*SE B*	β	*p*	95% CI		*B*	*SE B*	β	*p*	95% CI	
(Constant)	48.37	1.28		.000	45.85	50.90	29.59	4.20		.000	21.33	37.85
COVID-19-Specific												
**Timeframe**	**2.56**	**1.12**	**0.12**	**.023**	**0.36**	**4.77**	**2.89**	**1.15**	**0.15**	**.012**	**0.64**	**5.15**
**Lockdown exercise**	**0.82**	**0.28**	**0.15**	**.004**	**0.27**	**1.37**	**0.84**	**0.28**	**0.16**	**.003**	**0.29**	**1.39**
Socializing change	0.23	0.33	0.04	.489	-0.43	0.89	0.42	0.33	0.07	.214	-0.24	1.07
Financial hardship	-0.62	0.33	-0.10	.062	-1.28	0.03	-0.70	0.37	-0.11	.062	-1.43	0.04
Demographics												
Gender							-1.25	1.15	-0.06	.280	-3.52	1.02
Ethnicity							3.10	2.06	0.08	.134	-0.95	7.15
**Age**							**0.17**	**0.04**	**0.21**	**<.001**	**0.08**	**0.25**
Health							1.12	0.81	0.07	.164	-0.46	2.71
**Pre-COVID-19 exercise**							**0.51**	**0.19**	**0.15**	**.008**	**0.13**	**0.89**
Living alone							-3.45	1.77	-0.11	.052	-6.93	0.03
Ed. attainment							-0.34	1.14	-0.02	.768	-2.57	1.90
Household income							0.17	0.20	0.06	.382	-0.22	0.56
% Freelance							0.03	0.02	0.09	.120	-0.01	0.06
% Cont. income							0.01	0.02	0.03	.692	-0.04	0.06
% Cont. from art							0.02	0.02	0.05	.343	-0.02	0.05
Arts area							-0.45	1.56	-0.02	.774	-3.52	2.63
R^2	.053						.159					
Adjusted R^2	.042						.119					
ΔR^2	.053						.106					
F	4.87**						3.97***					
ΔF							3.53***					

Abbreviations: B, unstandardized beta; SEB, standard error of B; β, standardized beta; CI, confidence interval.
Note: *N*=353, **p* < .05, ***p* < .01, ****p* < .001, Gender = male, Ethnicity = white. Starting model: df (4, 352), VIF<1.03, Tol>0.98. Final Model: *df* (16, 352), VIF <1.76, Tol>0.57. Durbin-Watson = 2.05.

5D. Multiple linear regression modelling loneliness

	Model 1						Model 2					
	B	*SE B*	β	*p*	95% CI		*B*	*SE B*	β	*p*	95% CI	
(Constant)	4.87	0.20		.000	4.47	5.26	7.63	0.65		.000	6.36	8.91
COVID-19-Specific												
**Timeframe**	-0.32	0.18	-0.10	.070	-0.67	-0.26	**-0.39**	**0.18**	**-0.12**	**.027**	**-0.74**	**-0.04**
**Lockdown exercise**	**-0.13**	**0.04**	**-0.15**	**.004**	**-0.21**	**-0.04**	**-0.12**	**0.04**	**-0.14**	**.007**	**-0.20**	**-0.03**
Socializing change	-0.01	0.05	-0.01	.989	-0.10	0.10	-0.28	0.05	-0.03	.580	-0.13	0.07
**Financial hardship**	**0.13**	**0.05**	**0.13**	**.015**	**-0.03**	**0.23**	**0.13**	**0.06**	**0.13**	**.024**	**0.02**	**0.24**
Demographics												
Gender							-0.25	0.18	-0.71	.168	-0.59	0.10
Ethnicity							-0.36	0.32	-0.06	.263	-0.98	0.27
**Age**							**-0.02**	**0.01**	**-0.19**	**.001**	**-0.04**	**-0.01**
Health							-0.29	0.12	-0.10	.067	-0.47	0.02
**Pre-COVID-19 exercise**							**-0.07**	**0.03**	**-0.13**	**.026**	**-0.13**	**-0.01**
**Living alone**							**0.70**	**0.27**	**0.16**	**.011**	**0.16**	**1.24**
Ed. attainment							0.07	0.18	0.02	.685	-0.27	0.42
Household income							-0.04	0.03	-0.08	.186	-0.10	0.02
**% Freelance**							**-0.01**	**0.00**	**-0.12**	**.041**	**-1.01**	**0.00**
% cont. income							0.00	0.00	0.05	.456	-0.00	0.01
% cont. from art							0.00	0.00	-0.04	.423	-0.01	0.00
Arts area							0.37	0.24	0.08	.878	-0.44	0.51
R^2	.052						.191					
Adjusted R^2	.041						.153					
ΔR^2	.052						.140					
F	4.74**						4.97***					
ΔF							4.83***					

Abbreviations: B, unstandardized beta; SEB, standard error of B; β, standardized beta; CI, confidence interval.
Note: *N*=353, *p < .05, **p < .01, ***p < .001, Gender = male, Ethnicity = white. Starting model: df (4, 352), VIF<1.03, Tol>0.98. Final Model: *df* (16, 352), VIF <1.76, Tol>0.57. Durbin-Watson = 2.04.
