# Supplementary material for: The Effects of COVID-19 Lockdown 1.0 on Working Patterns, Income, and Wellbeing Among Performing Arts Professionals in the United Kingdom (April–June 2020)
Source: Front Psychol. 2021 Feb 10;11:594086. doi: 10.3389/fpsyg.2020.594086 (PMC7902701; doi:10.3389/fpsyg.2020.594086)
Supplement: Supplementary file 6 [file Table_6.pdf]

Spiro N, Perkins R, Kaye S, Tymoszuk U, Mason-Bertrand A, Cossette I, Glasser S, and Williamon A (2021), The Effects of COVID-19 Lockdown 1.0 on Working Patterns, Income, and Wellbeing among Performing Arts Professionals in the United Kingdom (April–June 2020), *Front. Psychol.* 11:594086. doi: 10.3389/fpsyg.2020.594086.

SUPPLEMENTARY TABLE 6 | Sources of support, *HEartS Professional Survey*, *N*=385.

	n	**%**
**Financial support (see Supplementary Figure 1, *HEartS Professional Survey*, question 5.9)**	**203**	
Yes	124	61%
No	79	39%
**If yes, where? (see Supplementary Figure 1, *HEartS Professional Survey*, question 5.10)**	**124**	
Colleagues	86	69%
Charities (arts-specific)	41	33%
Charities (general)	9	7%
Educational institutions	17	14%
Employers	34	27%
Family / friends	73	59%
Finance professionals (arts-specific) / advisors / banks / finance helplines	22	18%
Finance professionals (general) / advisors / banks / finance helplines	31	25%
Government-based agencies	49	40%
Insurers	3	2%
Teachers / coaches / mentors	8	7%
Trade unions	56	45%
Other	7	6%
**Health and wellbeing support (see Supplementary Figure 1, *HEartS Professional Survey*, question 5.12)**	**203**	
Yes	91	45%
No	112	55%
**If yes, where? (see Supplementary Figure 1, *HEartS Professional Survey*, question 5.13)**	**91**	
Colleagues	54	59%
Charities (arts-specific)	12	13%
Charities (general)	8	9%
Educational institutions	8	9%
Employers	9	10%
Family / friends	60	66%
Health professionals (arts-specific) / advisors / hospitals / helplines	6	7%
Health professionals (general) / advisors / hospitals / helplines	25	28%
Government-based agencies	5	6%
Insurers	1	1%
Teachers / coaches / mentors	10	11%
Trade unions	11	12%
Other	8	9%
