# Supplementary material for: The Effects of COVID-19 Lockdown 1.0 on Working Patterns, Income, and Wellbeing Among Performing Arts Professionals in the United Kingdom (April–June 2020)
Source: Front Psychol. 2021 Feb 10;11:594086. doi: 10.3389/fpsyg.2020.594086 (PMC7902701; doi:10.3389/fpsyg.2020.594086)
Supplement: Supplementary file 7 [file Table_7.pdf]

Spiro N, Perkins R, Kaye S, Tymoszuk U, Mason-Bertrand A, Cossette I, Glasser S, and Williamon A (2021), The Effects of COVID-19 Lockdown 1.0 on Working Patterns, Income, and Wellbeing among Performing Arts Professionals in the United Kingdom (April–June 2020), *Front. Psychol.* 11:594086. doi: 10.3389/fpsyg.2020.594086.

SUPPLEMENTARY TABLE 7 | Study 2. Indicative quotes for each sub-theme, presented according to five overarching themes.

Overarching theme 1 Lost or uncertain work and income

Sub-theme	Indicative quotes
1.1 Canceled, reduced, or changed work	My partner and I have both lost ALL our work for the foreseeable future as professional magicians. We have no income from those jobs now. Partner has got job as delivery driver and I'm doing admin to be able to survive. [Magician, female, age 47]
	I've slowly been building up work to earn a decent wage. Not only has work been cancelled that I had booked in until July but this will affect my work for the foreseeable future, long after the health situation is in control. [Actor, male, age 34]
	Most of my work is teaching in relation to theatre/performance in HE [higher education]. My teaching has been radically affected by the crisis. I was making performance with students; their assessment, teaching, and other practices have changed almost entirely. I have had to devote a lot of additional time to student support. [Theatre and live arts educator/researcher, female, age 52]
	It [lockdown] has obliterated my performance diary. I work with many smaller institutions and organisations who simply can't afford to pay for cancelled concerts and services. [Musician, female, age 22]
	I had five jobs lined up which have all been cancelled and there is no sign of work in the forceable [sic] future. My work in theatre and television involves working with people so there is no way that we can do that in isolation. [Actor, female, age 39]
	I have no performing work for the next two months at least. I have already lost several thousand pounds' worth of work, and expect this to increase. As a musician I only had one private student, choosing to focus more on performing work, and so as a consequence I have little to no income, as although I have managed to move this student online, the income generated is far from enough to survive on. I am effectively unemployed at the moment. [Performing arts professional unspecified, male, age 27]
	I[t] has caused the cancellation of all my family's work until further notice. [Actor, male, age 56]
	It has essentially ceased. Fundamentally, you cannot conduct without meeting other people in person. I was also furloughed by my primary employer. [Musician, male, age 32]
	I've lost all work. I've also lost an opportunity to audition for a job that was a real chance to progress my career and I don't know if I will ever get that chance again. [Actor, male, age 35]

		My work as an orchestral player has fallen off a cliff and I see no prospect of resuming my playing career any time soon, especially as I'm shielding with my husband who has complex health issues. I'm continuing with my teaching, but obviously only on line. [Musician, female, age 64]
1.2	Uncertainty for future work	Not sure how the industry will sustain itself or how quickly it will be able to recover once we get through this. [Actor, male, age 44]<br><br>My performing career as a freelancer has completely stopped, and there is no indication of when this might begin again. When it does pick up, there is no guarantee that I will be booked for anything. [Musician, female, age 35]<br><br>I feel…suspended. It feels like our profession as performing artists doesn't exist for the time being. And it feels it will take long and slow to restart. [Performing arts professional unspecified, female, age 45]<br><br>Thinking of a different occupation from the one I have devoted my life to since [childhood] is the most excruciating thing I have ever had to do. [Musician, female, age 39]<br><br>It's difficult to plan for future employment in theatre producing since no theatre can be created at this point and we don't know for how long this will continue. [Producer, female, age 39]<br><br>I feel despondent about how the performing arts is going to come back from this crisis. We have so many barriers to overcome: performing with others, performing in front of audiences, travelling etc. [Musician, female, age 50]<br><br>I feel stressed about the impact on friends and family and society as a whole. I feel we are in uncharted territory. [Musician, male, age 63]<br><br>Potential filming job postponed/cancel without payment. Theatre and film sets currently close, big drop in auditions and general lack of certainty about when work can go ahead again. [Actor, female, age 27]<br><br>Some days are wobbly. Just like in regular times, but now it seems bigger, more people are experiencing it, and it's got the weight of mass death and destruction attached. It's hard to imagine how we come out of this. [Actor, female, age 25]<br><br>I worry about the future and the impact this situation will have on the arts and culture sector. [Musician, male, age 38]<br><br>Anxious about the future of my company, the opera art form and the arts in general. [Musician, female, age 35]<br><br>The projects I was working on in late March 2020 have stopped. It's impossible to know whether they will recommence. It's impossible to know when planned projects might get under way, if they ever do. Many of the institutions I work for are in indefinite lockdown, from which some may never emerge.

	I'm a self-motivated person and can generate new projects for myself, but such projects all reflect the fact that I am a contributor to collaborative ventures, and such ventures are impossible to pursue at present and for the foreseeable future. [Actor, male, age 65]
1.3 Financial concerns	It’s been a disaster. An utter disaster. My industry disappeared overnight. Myself and my husband had two projects lined up (he had literally just started rehearsing his) all gone. All my freelance work gone. This will set us back at least £50k. And we have no control over it. [Performing arts professional unspecified, female, age 44]
	I don't think the current financial assistance properly takes into account those in my position who work as a freelance musician and who are also employed. My freelance earnings for the summer as a performing musician (and realistically probably well beyond) have been decimated (a £15k loss to date - at a time when which is usually the busiest in the year) and income from a regular teaching post is only paid over 10 months. I will therefore receive hardly any income in August and September but do not qualify for any help. [Musician, male, age 50]
	I have no work or income. I have had to temporarily live with my family. There are no new opportunities. [Dancer, female, age 22]
	All my concerts cancelled without compensation, composition commissions also cancelled without compensation. [Musician, female, age 27]
	All the work I had booked was cancelled within about 24 hours, in late March. As I freelance, and all my work relies on public buildings and institutions being open, I have had no work or income for the last 5 weeks. [Actor, female, age 54]
	All my live performances in the UK and abroad were cancelled pretty much overnight, totalling close to 40 performances. Unfortunately only a small handful of these were able to offer a modest remuneration for their cancelled concerts, some have rebooked for the following year, but most simply disappeared. [Musician, male, age 52]
	It has stopped us physically visiting schools and community centres, where most of our work occurs, and caused total loss of current income. [Multidisciplinary community practitioner, male, age 60]

Overarching theme 2 Constraints of lockdown working

Sub-theme	Indicative quotes
2.1 Challenges working or being at home	[I am] so so busy. I am trying to homeschool, keep fit, run the household, source food (so much more time consuming now), keep in touch with isolated relatives and friends as well as making sure my work does not suffer. I feel I do not have enough hours in the day. [Musician, female, age 44]<br><br>I have already struggled mentally getting into the office, but have avoided working at home as much as I could over the last few months, which helped. Having to work from home (where I don't yet have a proper set up) has been a big struggle again to get motivated, or to feel like I'm not letting people down by not doing my work. There has been a lot of admin to sort out in the last few weeks, and it has been hard to prioritise at the same time as looking after the only other member of my household (family) who was quite ill. Not knowing if the symptoms for myself and family are COVID-19 or something else was mentally difficult. It's also really difficult being restricted to one outing a day - I haven't managed to even get out for a walk around the block in the last 5 days as I've felt too rubbish. [Arts administrator, female, age 30]<br><br>My usual classroom teaching has shifted 100% online, but the workload has also increased 100 fold with added preparation time to redesign and deliver the remaining teaching materials and assessments online. My administrative workload AND time with students has also increased 100% as we are now expected to be logged on (and available online) continuously. Having to support students while we (the staff) are also going through a tough time, is particularly challenging… Mentally I feel cramped all the time. There is no head space to think creatively, especially for my research and writing. So the enjoyment from that aspect of my work has been scraped (temporarily I hope). I had to accept that I simply can't do everything right now and have put it on the back burner. Being confined indoors is also taking its toll mentally. Although the brighter spring weather helps with energy levels in the morning, every day now feels 'the same', or 'another day in the house'. I try to take short walks whenever I can and that can be particularly invigorating. But I don't manage it often. Working around the clock (to fit work and childcare) means that realistically there isn't that much time for exercise or relaxation, so my fitness is not what it used to be. Not having clear boundaries between work and family right now also makes me feel guilty when I try to 'squeeze' toddler time around work. It's hard to re-energise oneself to be a parent with work now having a physical (digital) presence inside the house, and conversely it's hard to switch to full professionalism when you are trying to do a zoom lecture with your little one screaming in the other room! [performing arts academic, female, age 42]<br><br>Continually having to change and adapt to the changing circumstances. Huge reliance on online platforms for all aspects of work including: communication, promotion, arts activity, mentoring, training. Reaching new audiences and opportunities in online work but hard to maintain momentum in all areas where there is no online replacement. [performing arts educator, female, age 30]<br><br>I have been depressed (diagnosed by doctor). This, I feel, is a result of an untenable increase in my teaching workload due to moving programme online and increased stress and pressure relating to it. My employers have expected me to shoulder the burden of this and have provided little to no meaningful support - financial or mental. This has in turn affected my creative output, reducing my ability to complete this work both due to the depression and to time pressures. The general impression the media gives of people in lockdown as having plenty of time to do nothing had exasperated this as the lockdown has increased rather than decreased the pressure I am under at work. [Actor and performing artist, female, age 47]

		I feel like I am not being creative enough with trying to come up with different opportunities for myself to create work for myself in quarantine as an actress. [Actor, female, age 24]
2.2	Struggles of online work	All of my work has moved online, and since I work mostly in a teaching capacity, I have struggled with changing lectures and workshops into this format and, as a freelancer lecturer cannot access training for these things (or would have to do training for free, whereas colleagues on a contract are paid for it). I have noticed this affects how I am able to engage with a group in a responsive and flexible way so my teaching has become teaching has become less energetic, less experimental and more didactic. [Producer, male, age 28]
		We are now doing a very small portion of our work online but it is not the same as doing it live. It's the difference between having a conversation with a stranger and recording a video for a stranger. [Actor, gender non-identified, age 49]
		Not having an audience is a huge challenge so the weekly community concert has kept us sane. [Community music practitioner, female, age 47]
		As I teach at home none of pupils can come to me anymore. With half of them I manage to start teaching on line, but as I am not expert in the remote technology it was a big challenge for me. I use my computer but my pupils (who are all school children age 8-18) use their telephones that results in restrict picture and weak quality sound, so I ended concentrating on simply continuity and general playing rather than paying attention to details like dynamics, articulation, phrasing and so on as I can't hear all this properly. Hence I concentrate mainly by encouraging the pupils and praising them so they have the motivation to continue playing and practicing. [Performing arts educator, female, age 73]
		I've mostly kept a handle on matters - keeping my playing up, housework, admin - but I spend FAR too much time staring at a screen and trying to teach via Zoom etc and when a student has poor wifi, it is enough to drive one to drink! [Musician, female, age 54]
		All teaching has moved online – this is a very different mode of working which is much more tiring and takes more preparation. It is also harder to coach performance and discuss musical matters in detail because of technical difficulties/broadband speed with those communicating with. [Musician and producer, female, age 31]
		Trying to work online but it's just not the same. [Performing arts professional unspecified, male, age 49]
2.3	Difficulties maintaining skills or practices	I am unable to sing in choirs. I am unable to practise the organ, as the instrument I use is in a church, and all churches are currently closed. [Musician, male, age 38]
		I cannot practice anymore (my dance style can't be practised in my home as it's too loud and needs too much space). [Dancer, female, age 32]
		[It's a] really bleak period: hard to be creative. [Musician, male, age 22]

		Because of my age I am forced to self-isolate. I am alone in my flat and although I am very busy with online streaming with friends and other comedians, I am hungry for human contact. One begins to forget that there are others out there and that the world is not just you alone. I am a writer and a communicator and I feel stifled. [Burlesque/cabaret performer, female, age 86]<br><br>I am certainly concerned about maintaining my own professional playing (piano/organ) skills. [Musician, female, age 31]<br><br>I'm getting rusty! [Actor, male, age 65]<br><br>All the hard work of rehearsals, has unfortunately now come to nothing. The biggest worry is how dancers can keep truly fit, whether directors will rehabilitate us thoroughly? [Dancer, gender not disclosed, age 21]
2.4	Caring responsibilities	Trying to work from home, homeschool and look after 2 children and look after my own fitness and mental well being has been my biggest challenges. [Performing arts professional unspecified, female, age 39]<br><br>Our trio will meet soon for rehearsals of our August and September programmes in the garden (keyboard with extension lead) which we are looking forward to. On the positive side, we may be able to update or add to our tech skills by recording for radio transmission and videoing some of our performances. However, all three of us, being female, have found we have less time for practice whilst looking after our families. Without regular (5+ per month) performances, we are possibly becoming de-skilled. [Musician, female, age 59]<br><br>I am spending little time managing my own health and wellbeing as I have taken on the care of a vulnerable relative who needs shielding during the crisis, who's [sic] needs are demanding. [Performing arts professional unspecified, female, age 45]<br><br>What's become clear too, is that in my organisation, the board are dealing with that loss of control of their own lives and trying to impose control on me and my staff. Endless demands, total lack of understanding of how we are at home, trying to work, during a time of crisis, when we have families, young children, loved ones, to take care of. The shift has been seismic and it's not always understood by the patrician gatekeepers and power holders. [Producer, male, age 50]<br><br>I have lost all my playing and directing. I have had to switch to online teaching on school chosen systems at days notice, while the class teachers have had training and a gap before they started. The children are home and need supervision. It's been hard! (Musician, female, age 48)<br><br>My anxiety has increased about future work and the challenges of maintaining work whilst being at home full time with all my children. [Musician, female, age 35]

Overarching theme 3 Loss, threat, and vulnerability

Sub-theme	Indicative quotes
3.1 Reduced social connections	A freelance musician is by their very nature a sociable and well connected person, and so many are struggling with the sheer contrast of going from seeing other like-minded individuals everyday, to not seeing them at all for a considerable amount of time. [Musician and arts administrator, male, age 20]
	The biggest impact is for my choir. The well documented benefits of singing in a choir are so important in my life in normal times that while I am running online sessions and they are fun, up to a point, they can't have the amazing buzz and social interaction of proper rehearsals. [Musician, female, age 53]
	I live alone and am usually happy with this but the lack of social contact since lockdown has been very difficult. [Visual artist and performing artist, female, age 45]
	I miss performing and interacting with my colleagues. [Musician, female, age 40]
	I feel separated from people, particularly from not teaching. It is isolating and lonely. [Dancer, female, age 22]
	Feeling very insular and can't see an end to it. [Actor, male, age 65]
	I miss the camaraderie of rehearsing and performing. I miss the laughter and the fun. I miss having the sound of an orchestra around me. [Musician, female, age 59]
3.2 Lack of support and vulnerability	Working freelance in the performing arts is always a precarious business. You are constantly trying to make 'things' happen with very little financial gain. You write, you attend auditions and workshops, you establish theatre companies and apply for funding ... None of these activities are paid. You might get a piece of writing acknowledged in a competition, you might get the part, you might be awarded some money. You put out in the community to provide opportunities for others ... You provide invisible earnings. Now it is just harder. Almost impossible. [Actor, female, age 62]
	Playing work is zero and is on rolling cancellation as the lockdown continues. Freelance orchestral work is now being offered on a 'pencil it in' basis so that if the lockdown isn't over by then the work can be rubbed out by the orchestras with no question of cancellation fees - understandable, they're potentially in financial trouble too. From choice my teaching has been in a state school with students whose parents have little money at the best of times. Lessons were heavily if not totally subsidised by the school so even pre-crisis these parents were not in a position to pay for online lessons. I am therefore now doing a small amount of free online teaching to keep them interested and motivated (I hope!). [Musician, female, age 59]
	It makes me question the point of being in the arts when there is little to no governmental or even general public support. [Musician, female, age 22]

Having contracted COVID-19 I have certainly been fearful and anxious. I felt like I was walking into a death trap working in a care home as the PPE [personal protective equipment] is not in wide use and getting COVID-19 seems to be ‘it may or may not be serious’ scenario. I have only received SSP [statutory sick pay] while I have been off work. I have savings so this hasn’t massively worried me but does feel a bit of a kick in the teeth after putting myself and family at risk by going to work. [Music therapist, male, age 34]

Because I have PAYE [Pay As You Earn] income from a university job that is more than 50% of my earnings (if my income from performing is defined as ‘profit’) then I will not qualify for government support. I am very concerned that this could become a very serious situation for me. [Musician and researcher, female, age 45]

[There] seems to be an expectation that Musicians cheer everyone up for free - it’s our livelihood and we’ve just lost most of it! [Musician and performing artist, female, age 35]

Unfortunately the governments 'self employment scheme' doesn't help me. I am a recent graduate and have a net profit of below 50%. Its looking like universal credit would is my only option but as I'm under 25 this will amount to less than £80's a week and can take up to 5 weeks to process. While there are government schemes in place I feel like I am falling through the cracks. This has significantly impacted my mental wellbeing and financial security. [Actor, female, age 24]

As a freelancer I put all my work through a limited company. Most of my contracts have been cancelled or postponed indefinitely. I am not eligible for any govt [sic] support like several colleagues. [Sound engineer, male, age 32]

3.3 Sense of loss or grief

How to have a sense of self-worth when your job does not exist anymore? [Writer, TV, and radio presenter, female, age 41]

I am also in a kind of mourning for the industry which I know will never be the same. And there is a huge sense of grief and foreboding for all of my peers and the many wonderful organisations that I work with. [Arts administrator, female, age 32]

Music is my life, I am lost without it. I earn very little money wise and only perform part time usually. It is also my hobby and most of my social life too, so I miss it terribly. [Performing arts professional unspecified, female, age 61]

I have felt my emotions leap from euphoria- the opportunity to see a slower calmer environment. Quieter more peaceful times. Noticing nature, spring flowers, birds to a sense of desperate grief. Grief for the loss of life, but also grief for the world as we struggle to make sense of what is happening! [Musician and performing artist, female, age 55]

I am experiencing anxiety for the first time in my professional life. I have lost my whole sense of self-identity because I am no longer working. [Performing arts professional unspecified, female, age 44]

		When do get to go outside ourselves, experience the lives of others, in a shared space – the one that helps us empathise again in this broken world? What I'm describing is a type of grief. A loss. [Producer, male, age 50]<br><br>If you play 70-80 concerts each year and suddenly over half of these disappear there is a sense of loss, both emotionally and physically which is hard to replace. [Musician, male, age 52]<br><br>My theatre/TV industry went down overnight I felt angry and sad regarding this, I have felt grief for the loss of our industry and how that feeds my soul. [Actor, musician, performing artist, and producer, female, age 52]
3.4	Concern for beneficiaries/loved ones	I run a music project with vulnerable/excluded young people. We have had to 'pause' the project for 3 months. It affects my income, but more so, it upsets me that we cannot deliver a meaningful project to young people who need it. [Community music practitioner, female, age 53]<br><br>[I am] very afraid and worried for my elderly mother [for] whom I am primary carer. Having to also manage and deal with the stress and distress of my partner (also in the arts and out of work = also no income!) has added to my own. [Performing arts professional unspecified, male, age 42]<br><br>I work for a music charity as Musical Director, working in prisons and with ex-offenders. That work has completely stopped, and our 14 musicians in residence are not delivering weekly sessions with 500+ participants. The work is well documented as having a positive impact on well being and hope for the future, and a significant reduction in reoffending. I am worried about the impact of the current crisis on our outcomes, and the well being of participants. [Music director, male, age 42]<br><br>Our main challenge is the fact that a grandson works in a hospital pharmacy department and his girlfriend (a Spanish national) is a nurse with the same Health Board in Wales. We are, understandably, very concerned for their welfare. [Actor, female, age 74]<br><br>My mum is in lockdown on her own and I am anxious about her. [Journalist, musician and performing artist, female, age 32]<br><br>The biggest challenge though is not being able to go out and help as much as I would like due to having to shield my partner who already has a serious lung condition. [Musician, female, age 59]<br><br>I miss working in person with children and teenagers and many of them are struggling so I am trying to help them along as best as possible. We have all lost support. [Musician and educator, female, age 48]

Overarching theme 4 Detrimental effects on health and wellbeing

Sub-theme	Indicative quotes
4.1 Anxiety	I've had increased anxiety and that is not something I commonly suffer from. I feel very stressed not only about the future of my career, but also my health, my finances, and the wellbeing of my family and friends. [Musician, female, age 26]
	Low health, feeling trapped, a lot of anxiety. [Musician, non-binary, age 21]
	My anxiety about leaving the house in the car, or on foot to a public place is considerably higher. I have noticed far more drastic physiological symptoms relative to anxiety and panic. [Arts administrator, female, age 24]
	I have depression and anxiety and the current situation has meant my body has been extremely anxious and I am unable to calm it down. [Dancer and performing artist, female, age 23]
	Anxiety, fear, unable to concentrate or do basic things on most days. [Actor, female, age 54]
	It is impacting on my mood and general well being. I am aware of underlying stress and anxiety causing mild hypermania, insomnia, sadness, and anger. [Music therapist, female, age 53]
	Very anxious about money and health, unable to visit friends and family. [Performing arts professional unspecified, male, age 27]
	Made me very stressed and panicked. [Educator, female, age 50]
	I have had days when I have felt very low, and days when my anxiety has been very high. The prospect of the government deciding to limit our freedom further is filling me with a lot of fear. [Musician, male, age 30]
	The lack of income and sense of professional identity has caused much anxiety and stress. The loss of choral singing has also had a hugely negative impact [on my] mental well-being. [Performing arts professional unspecified, female, age 36]
	I am experiencing an undercurrent of survival anxiety. [Actor, gender non-identified, age 49]
4.2 Low or unstable mood	I am mentally breaking down every day. I am trying to care for my 6 year old and keep my business afloat with no income on an open ended time span. I am depressed and struggling every moment of every day. It is horrendous and has destroyed my life. [Arts administrator, female, age 46]

I have had spells of depression since the beginning of the pandemic as I have been forced to face the prospect that I will be unable to do the job I love and have trained for in the future. [Musician, female, age 28]

The removal of any orchestral playing and pit work that I do has destroyed my mental stability and hugely increased my anxiety levels. I enjoy my salaried role (non music related), but it is my performing hobby (unpaid and paid) that keeps me mentally stable. I see no prospect of this starting up any time soon and that simply makes things worse. Practising alone at home simply doesn't have the same impact. [Performing arts professional unspecified, female, age 57]

Am cycling more to maintain fitness, but struggling with depression. [Musician, male, age 52]

I am feeling quite anxious and a bit depressed as I have dedicated the majority of my life to honing my skill as a classical violinist, and I cannot get a job at a supermarket. I have little motivation to practice, as I don't really see the point anymore. [Musician, female, age 32]

Am very up and down. I'm fortunate to live in a nice part of the country with easy access to open spaces and farm shops, and although my income is seriously reduced as a result of the pandemic, I'm still able to earn money from teaching online. Nonetheless, it's an utterly depressing situation and I fear for the future of the arts in this country, as well as my own personal future. We've lost so much in such a short space of time, and the fear engendered in the general population won't be quickly or easily overcome. [Musician and performing artist, female, age 44]

4.3 Poorer physical health

As an energetic performer, collaborative researcher, teacher and someone who uses the local gym a few times a week I am using my brain energy a lot but my physical health is suffering as the local walk isn't really enough to keep my body in shape. The lack of exercise in a pool is starting to impact on how I feel both physically and mentally. My sleep has been affected. I no longer fall asleep easily and I have a few times woken up from troubling dreams related to the current situation. [Musician, female, age 40]

Eating more than usual. Drinking more than usual. Putting on some weight. Unable to be outdoors as much as usual. Not able to take part in a weekly group Tai Chi Class. [Actor, male, age 59]

Struggled to sleep after having contract terminated. [Actor, female, age 27]

I suffer with a pre-existing heart condition, for which I was on a waiting list to have surgery. My symptoms have been gradually worsening and I've been stuck not knowing what to do. I think for people with existing conditions it's going to take the NHS a long time to catch back up. [Arts administrator, female, age 26]

My job is very focused on health and well-being of dancers so I found I worked extra hard to support them at the moment, creating resources, suggesting things for their wellbeing but in so doing overworked myself and my health began to deteriorate. [Researcher and educator, female, age 34]

		I'm putting on weight due mainly to lack of exercises. [Musician, male, age 55]
		Increased headaches and jaw and neck tension in week one and two. Lack of sleep, strange dreams. Fear of putting on weight. [Actor, female, age 27]
		I am, for the first time, suffering anxiety. Lack of sleep, inertia, lack of dedication and so on. Eczema is thus up, breathing is shallow and so on. [Musician, female, age 35]
4.4	Lack of motivation or focus	The real challenge is keeping yourself motivated/occupied. With all work being cancelled for the foreseeable future, it's difficult some days to know what to do with yourself. [Musician and researcher, male, age 27]
		I feel moody, anxious, I can hardly push myself to do something productive. Everything seems pointless and I feel forgotten. [Musician and dancer, female, age 22]
		It's hard to stay positive and motivated, and hard not to feel like my entire job is a complete waste of time. [Arts administrator, female, age 34]
		Living alone, I am finding it hard to stay motivated to do anything, both creatively and normal day to day tasks. [Director, female, age 55]
		I have struggled very much with motivation to do anything, including practice. I would love to be able to take the opportunity to use all of this extra time effectively and be super productive, but instead I feel guilty and anxious for feeling unable to do so. I don't know when things will be back to normal or how I will fill my time until then, or how I'll be able to motivate myself to be productive in that time. I could probably find more things to be doing work-wise but I'm struggling even to practise and keep up with my own and everyone else's expectations of what I should be doing. [Musician, female, age 23]
		My ability to carry out research has all moved online, and performances and rehearsals with others are cancelled. A few online rehearsals proved how platforms such as Zoom don't allow for synchronised playing together. As such, only recording, practicing, and planning can happen now, but I'm unmotivated to do even that since no future performance or meeting acts as a goal. [Musician, male, age 26]
		I am actually deriving a lot of satisfaction by using online tools to help my students and colleagues but I feel a bit disheartened and am finding it hard to keep myself motivated to practise for concerts that are now some way in the future. [Musician, female, age 50]
		Lack of focus, motivation, constantly tired and often stressed. [Performing artist and writer, female, age 59]

Overarching theme 5 Professional and personal opportunities

Sub-theme	Indicative quotes
5.1 Coping fine or living more healthily	I am healthy and have more time to rest and do sports, so I am probably a bit healthier than before. No travelling and good night sleeps help as well. I feel fine! [Musician, female, age 34]<br><br>Health is fine. I am getting more exercise than before. [Musician, male, age 61]<br><br>My health and wellbeing has been OK as I have learned to value my choices of food purchases more and to cut food waste to zero, and also to eat healthier meals including some produce I grow on my allotment. It has also been immensely beneficial to my mental health for me to go out and deeliver [sic] some food parcels of foods that I have put together for a couple of friends who due to disability are unable to go out and access shops as regularly as they could before the lockdown. [Performing arts professional unspecified, non-binary, age 57]<br><br>I am cooking much more and eating regular meals so I think my health and well-being are improved! [Musician, female, age 72]<br><br>It [lockdown] has not had an impact in general. [Community music practitioner, male, age 30]<br><br>As a writer/reviewer/editor/researcher I am used to a solitary working lifestyle! [Writer, reviewer, editor, and researcher, male, age 48]
5.2 More time and less pressure	I have worked non-stop since 16 [years old] and this has made me step back and take a break to spend time with my family. It's a horrible situation and it's scary to think what could happen but I'm not letting the worry consume my life. I am lucky to not have to worry about taking a break from work, not having any earnings for the short term. [Performing arts professional unspecified, female, age 43]<br><br>Two of our children are at home with us, and there is more time to spend with them and on activities that have long been neglected. [Musician, male, age 53]<br><br>I feel a lot less stressed overall, and enjoy having less pressure in my day to day life. I have been taking more time for wellbeing activities like meditation and I am really enjoying it. [Dancer, female, age 29]<br><br>During lockdown I have been able to stretch and practice yoga every day (through Yoga with Adriene, online classes, college classes etc) and am training for a marathon. This has been a godsend for my mental health, allowing me to take quiet time in a busy (and very nosey!) household and connect with nature in the outside world. Running has sometimes given me a chance to have proper conversations with my boyfriend who I am isolating with, which is a challenge when we have been surrounded by family. In general, lockdown has allowed me to slow down and assess my goals and needs. I have also managed to work on an ongoing issue with my back which I felt was blocking me. I feel guilty saying this, but I have quite enjoyed this period as I am

	normally so stressed and busy, although I do miss performing and have had some sad days when I think about the projects I would have been doing right now. [Musician, female, age 24]<br><br>I think I may benefit from working less by having more 'me time', doing online fitness and yoga classes more often than I would normally had time for. Setting up some meditation goals too. [Arts administrator, female, age 39]<br><br>My quality of life has improved as I [have] had more time at home to be in the garden and on lovely country walks. Plus I'm having more quality time with my husband. [Musician, female, age 54]<br><br>I feel a lot less stressed overall, and enjoy having less pressure in my day to day life. I have been taking more time for wellbeing activities like meditation and I am really enjoying it. [Dancer, female, age 29]
5.3 New possibilities and activities	I usually spend a great deal of time in the future. Researching, preparing, scheduling rehearsals, nurturing and collaborating. There is no surity [sic] of anything in the future. Even agreed projects 3 years away will shunt or disappear. I have gone in to [sic] myself to re-evaluate everything. Love and care has overridden. [Musician, male, age 58]<br><br>I am using the time to research as an artist my skills and abilities. I am developing new projects and paths of research. [Arts administrator, performer and educator, female, age 30]<br><br>I am actually caring quite well for my wellbeing, doing other hobbies. An opportunity from this chaos is being able to be taught classes by amazing teachers from all over the world, albeit through a screen. [Dancer, gender not disclosed, age 21]<br><br>In a way, it has been nice to have time to recuperate and spend some time planning new projects, but everything has felt very limited. I'll be taking this opportunity to begin recording projects, and working with my teacher and others in our department to create an 'online studio' to showcase existing recordings and to make new ones, solos or chamber music, and we will also be working with a composer who used to be in the department. Also working with Live Music Now I plan to record videos for them to post/send. One of my composer friends has been writing isolation themed miniatures for friends to record and post online, which I will be completing soon. [Musician, female, age 23]<br><br>I have plenty of time to practise, and learn new repertory. I am able to embark on a 3-volume pedagogical anthology, commissioned by an American publisher. [Musician and researcher, male, age 72]
5.4 Enhanced social connections	It has made me feel better connected with the wider industry – I've had to do more online searching and have come across great resources and networks as a result. Working in the arts feels incredibly collaborative right now. My work is currently the area of my life in which I feel less anxious, most supported and the least alone. [Arts administrator, female, age 24]

	My arts and cultural work has moved mainly extremely local, i.e. a horseshoe of three streets for whom we perform weekly concerts outdoors, socially distanced. The local community is much stronger for it – everyone attends regularly, there is much appreciation of the occasion to see others, the appreciation of the music is huge even though many who turn up are unlikely to have ever entered a concert hall before. [Community music practitioner, female, age 47]
	I'm in the fortunate position of being able to decamp to my partner's house out of London, where we support each other. [Educator, male, age 68]
	Emotionally I feel pretty balanced. Very lucky to have a small family (who I actually like) plus my brother staying with us so there is plenty of company and laughter about the house. Two young children don't allow me time to consider how it must feel to be bored with nothing to do so that is a positive! My wife is a key worker so I am doing more of the homeschooling and keeping them entertained than before. It can be very challenging and exhausting on the tricky days but I love that we are getting the chance to bond ever more closely and getting to share lots more precious moments. [Musician, male, age 43]
	So I would say things definitely could be worse and we are coming through this stronger and more bonded as a family. [Choral director, female, age 35]
	The positive side is that there has been time for planning and research; and I have been part of phoning support groups. There has been plenty of communication online, and Zoom meetings have been useful, both for business and planning, and for social groups. [Musician, male, age 68]
5.5 New skills	Opportunities: finding new ways of doing things, applying creativity and new technological solutions. [Arts administrator, male, age 30]
	It has forced me to use new media and overcome challenges, learn a lot of new skills and adapt. I needed to develop a lot of patience. [Music therapist, female, age 51]
	It has made me think about, and learn to do things in different ways, stimulating creative ideas, and enabled me to challenge others and myself to more creativity, and learn another instrument. [Music therapist, female, age 63]
	IT [Information Technology] skills improved. [Arts administrator, female, age 46]
	However am becoming far more savvy online. Gave my first livestream concert. [Sound designer and performing artist, female, age 54]
	Learning how to use new technologies and system to continue working. [Arts administrator, female, age 40]
