# Supplementary material for: The Effects of COVID-19 Lockdown 1.0 on Working Patterns, Income, and Wellbeing Among Performing Arts Professionals in the United Kingdom (April–June 2020)
Source: Front Psychol. 2021 Feb 10;11:594086. doi: 10.3389/fpsyg.2020.594086 (PMC7902701; doi:10.3389/fpsyg.2020.594086)
Supplement: Supplementary file 8 [file Data_Sheet_1.pdf]

HEartS
Health, Economic, and
Social impact of the ARTs

1. Welcome and consent
2. Demographic questions
3. Coronavirus (COVID-19)
4. Work
5. Income and support
6. Open response
7. Health and wellbeing
8. Social connections
9. Further information

HEartS Professional Survey

Note

Responses were collected through the Qualtrics online platform over a period of ten weeks: 1 April – 15 June 2020.
Previously published scales are named here but not reproduced in full.

Dataset

Williamon A, Spiro N, Kaye S, Tymoszuk U, Mason-Bertrand A, and Perkins R (2021), HEartS Professional Survey: Charting the Effects of COVID-19 Lockdown 1.0 on Working Patterns, Income, and Wellbeing among Performing Arts Professionals in the United Kingdom (April–June 2020), *Dryad*, Dataset, doi: 10.5061/dryad.s7h44j14z.

Report

Spiro N, Perkins R, Kaye S, Tymoszuk U, Mason-Bertrand A, Cossette I, Glasser S, and Williamon A (2021), The Effects of COVID-19 Lockdown 1.0 on Working Patterns, Income, and Wellbeing among Performing Arts Professionals in the United Kingdom (April–June 2020), *Front. Psychol.* 11:594086. doi: 10.3389/fpsyg.2020.594086.

Correspondence

Aaron Williamon, aaron.williamon@rcm.ac.uk

Funded by the Arts and Humanities Research Council (AHRC)
HEartS: Grant Ref. AH/P005888/1 | United Kingdom
HEartS Professional: Grant Ref. AH/V013874/1 | United Kingdom

1. Welcome and consent

Welcome to the survey

Thank you for considering taking part in our research. We are interested in your professional creative, artistic, and cultural pursuits, and your wellbeing. By completing this survey you will be providing valuable insight into the impact of the current public health situation on the work, livelihood, and wellbeing of those who work in the arts and cultural areas.

This survey is for people who:

- currently live in the United Kingdom
- work in the arts (i.e. part of your livelihood comes from work in the arts and cultural areas)
- are 18 years old or over

For surveys in English for professional artists living in Australia, Canada, New Zealand, and the USA, please click on your country. If you live elsewhere and would like to take part, contact the research team at hearts@rcm.ac.uk.

For now, please complete this survey only once.

Please note, you may leave and return to complete the survey later on the same device.

Consent

Your participation in this research is voluntary, and you may withdraw from the study at any time if you wish.

What will we do with your data?

The data you provide will be anonymous (separated from your name) and confidential (not disclosed to anyone else). The data will be stored securely at Imperial College London and the Royal College of Music according to the Colleges' Data Retention Policies, and used as part of the HEartS project which is funded by the UK's Arts and Humanities Research Council to explore the health and social impact of the arts. We plan to publish portions of our data set, reports, articles, and presentations based on our findings, but you will not be identifiable from the data.

Who should I contact if I have queries about the research?

If you have any queries or concerns about the research, you can contact our research team at hearts@rcm.ac.uk. The project has been reviewed and approved by the Research Ethics Committee of Conservatoires UK.

We will be asking you questions about your experiences with the current public health situation arising from the Coronavirus (COVID-19), your mental health and wellbeing. Therefore, at the end of the questionnaire, there will be links to sources of information and support on these issues.

1.1 Please confirm that this is the first time you are completing this survey:

❑ Yes, this is the first time I am completing this survey.

1.2 Please confirm that you are living in the UK:

❑ Yes, I am currently living in the UK.

1.3 Please confirm that you are 18 years or older:

❑ Yes, I am 18 years or older.

1.4 Please confirm that you give your informed consent to participate in the study:

❑ Yes, I consent to participate.

1.5 Are you willing to take part in future research?

If yes, leave your email address here: ___________________________

Please note: Your email address will be stored separately from your responses to the survey and will only be used to contact you with an invitation to take part in future research.

1.6 Please re-enter your email address: _________________________

2. Demographic questions

2.1 In which region do you live?

❑ Northern Scotland
❑ Southern Scotland
❑ North East
❑ North West
❑ Yorkshire and the Humber
❑ East Midlands
❑ West Midlands
❑ East of England
❑ South East
❑ South West
❑ London
❑ North Wales
❑ South Wales
❑ Northern Ireland

2.2 How old are you?

(Please write in numbers, e.g. 42) _________

2.3 I identify myself as

❑ Male
❑ Female
❑ Would rather not say
❑ Other (please define) _____________________________________

2.4 I classify myself as (please tick all that apply):

This list is adapted from the Office for National Statistics. Different countries and groups use different labels, so please add your own if you wish.

❑ White - English / Welsh / Scottish / Northern Irish / British
❑ White - Irish
❑ White - Gypsy or Irish Traveller
❑ Any other White Background
❑ Mixed / Multiple ethnic groups - White and Black Caribbean
❑ Mixed / Multiple ethnic groups - White and Black African
❑ Mixed / Multiple ethnic groups - White and Asian
❑ Any other Mixed / Multiple ethnic background
❑ Asian / Asian British - Indian
❑ Asian / Asian British - Pakistani
❑ Asian / Asian British - Bangladeshi
❑ Asian / Asian British - Chinese
❑ Any other Asian background
❑ Black / African / Caribbean / Black British - African
❑ Black / African / Caribbean / Black British - Caribbean
❑ Any other Black / African / Caribbean background
❑ Arab
❑ Any other ethnic group

2.5 In which of the following arts and cultural areas do you work professionally? (please tick all that apply)

*By **professional** we mean that some part, or all, of your livelihood comes from work in one or more arts and cultural areas.*

*At this point, we want to know the general areas in which you work and **not what you do (i.e. your particular roles)** within those areas. We will ask later about what you do.*

❑ Crafts
❑ Decorative arts
❑ Literature
❑ Music or sound arts
❑ Performing arts
❑ Visual arts

2.6 At what age did you **begin working professionally** in your arts and cultural area(s)?

If you work in more than one area, count from the one you started first.

(Please write in numbers, e.g. 42) ________

2.7 Are you currently studying?

❑ No
❑ Yes

If yes

2.8 Are your studies related directly to your work in the arts and cultural areas?

❑ No
❑ Yes

If yes

2.9 What is your course? _________________________

2.10 What is your institution? _________________________

2.11 What is your mode of study?

❑ Full-time
❑ Part-time

2.12 What is the highest educational and/or vocational qualification you have thus far obtained?

❑ Secondary qualification (e.g. high school diploma)
❑ Tertiary / higher/ further qualification (e.g. bachelors degree)
❑ Advanced qualification (e.g. masters, PhD, DMA, DMus degree)

2.13 With whom do you live? (please tick all that apply)

❑ On my own
❑ Residential care setting
❑ My spouse or partner
❑ Children
❑ Other family
❑ Friends or house share
❑ Other
❑ Would rather not say

3. Coronavirus (COVID-19)

This section explores your experience of the public health situation arising from the Coronavirus (COVID-19).

3.1 Have you had or do you currently have Coronavirus (COVID-19)?

❑ Yes, I have tested positive
❑ I think so, I have (or have previously had) symptoms
❑ Not that I know of

3.2 Are you self-isolating?

❑ Yes, I am currently self-isolating alone
❑ Yes, I am currently self-isolating with others
❑ Not currently, but I have self-isolated and mainly did so alone
❑ Not currently, but I have self-isolated and mainly did so with others
❑ No, I am not self-isolating and haven't yet done so

If yes

3.3 How many days have you been (or were you) in self-isolation? Count your first day as Day 1.

{Slider | Units: days | Range: (0 days) – (112 days)}

3.4 In the last month, has the public health situation affected how many people you spend time with **in person**?

*By **in person** we mean that you are with them in their physical presence, including family members, house mates, friends, work colleagues, etc.*

❑ Substantially fewer people, in person
❑ Far fewer people, in person
❑ Fewer people, in person
❑ No change
❑ More people, in person
❑ Far more people, in person
❑ Substantially more people, in person

3.5 In the last month, has the public health situation affected how many people you spend time with **electronically**?

*By **electronically** we mean that you are with people online, on social media, or on the phone, including family members, house mates, friends, work colleagues, etc.*

❑ Substantially fewer people, in person
❑ Far fewer people, in person
❑ Fewer people, in person
❑ No change
❑ More people, in person
❑ Far more people, in person
❑ Substantially more people, in person

3.6 In the last month, how has the public health situation affected how **lonely** you feel?

❑ Much more lonely
❑ Quite a lot more lonely
❑ A little more lonely
❑ No change
❑ A little less lonely
❑ Quite a lot less lonely
❑ Much less lonely

3.7 In the last month, how has the public health situation affected how **anxious** you feel?

- ❑ Much more anxious
- ❑ Quite a lot more anxious
- ❑ A little more anxious
- ❑ No change
- ❑ A little less anxious
- ❑ Quite a lot less anxious
- ❑ Much less anxious

4. Work

We would like to know more about your work portfolio.

4.1 Within the arts and cultural areas in which you work professionally, please tell us in what field(s) you work.

{Logic based on 2.5}

Crafts and Decorative arts (please tick all that apply)

- ❑ Ceramics
- ❑ Cooking
- ❑ Floristry
- ❑ Furniture making
- ❑ Gardening
- ❑ Glass working
- ❑ Interior design
- ❑ Jewellery making
- ❑ Metal working
- ❑ Pottery
- ❑ Rug / carpet / tapestry making
- ❑ Sculpture
- ❑ Textiles
- ❑ Wood working
- ❑ Other (please specify) ________________________________
- ❑ Other (please specify) ________________________________

Literature (please tick all that apply)

- ❑ Film / television scripts
- ❑ Illustrations / cartoons / comic strips
- ❑ Novels / novellas
- ❑ Plays
- ❑ Poetry
- ❑ Short stories
- ❑ Other (please specify) ________________________________
- ❑ Other (please specify) ________________________________

Music or sound arts (please tick all that apply)

- ❑ Classical
- ❑ Dance (including house / techno)
- ❑ Folk / country
- ❑ Jazz / blues / R'n'B
- ❑ Musical theatre
- ❑ Pop / rock
- ❑ Rap / hip-hop
- ❑ Video games
- ❑ Other (please specify) ________________________________
- ❑ Other (please specify) ________________________________

Performing arts (please tick all that apply)

- ❑ Acrobatics
- ❑ Acting
- ❑ Dancing
- ❑ Circus arts / physical theatre
- ❑ Stand up comedy
- ❑ Gymnastics
- ❑ Magic
- ❑ Musical theatre
- ❑ Puppetry
- ❑ Other (please specify) _______________________________
- ❑ Other (please specify) _______________________________

Visual arts (please tick all that apply)

- ❑ Drawing / illustration
- ❑ Ceramics
- ❑ Design
- ❑ Film / video making
- ❑ Painting
- ❑ Photography
- ❑ Printmaking
- ❑ Sculpture
- ❑ Other (please specify) _______________________________
- ❑ Other (please specify) _______________________________

4.2 Within your arts and cultural work, what are your main activities? (please tick all that apply)

- ❑ Appraising / assessing / evaluating / reviewing
- ❑ Composing / choreographing / designing / making / writing
- ❑ Conducting / directing / producing
- ❑ Curating / editing / presenting / technical supporting
- ❑ Managing / promoting
- ❑ Performing
- ❑ Researching
- ❑ Teaching / coaching / workshop leading / mentoring
- ❑ Other (please specify) _______________________________
- ❑ Other (please specify) _______________________________

4.3 Indicate the percentage of time you would **normally** devote to each of these activities **in your arts and cultural work portfolio**.

Sum should add to 100%.

{Logic based on 4.2}

Appraising / assessing / evaluating / reviewing
{Slider | Units: percentage}

Composing / choreographing / designing / making / writing
{Slider | Units: percentage}

Conducting / directing / producing
{Slider | Units: percentage}

Curating / editing / presenting / technical supporting
{Slider | Units: percentage}

Managing / promoting
{Slider | Units: percentage}

Performing
{Slider | Units: percentage}

Researching
{Slider | Units: percentage}

Teaching / coaching / workshop leading / mentoring
{Slider | Units: percentage}

Other 1 (please specify) ___________________________
{Slider | Units: percentage}

Other 2 (please specify) ___________________________
{Slider | Units: percentage}

4.4 Considering the time you would normally devote to each of these activities, how has the time spent doing each changed **since the start of the current public health situation**?

{Logic based on 4.2}	Much less	Quite a lot less	A lot less	No change	A little more	Quite a lot more	Much more
Appraising / assessing / evaluating / reviewing	❑	❑	❑	❑	❑	❑	❑
Composing / choreographing / designing / making / writing	❑	❑	❑	❑	❑	❑	❑
Conducting / directing / producing	❑	❑	❑	❑	❑	❑	❑
Curating / editing / presenting / technical supporting	❑	❑	❑	❑	❑	❑	❑
Managing / promoting	❑	❑	❑	❑	❑	❑	❑
Performing	❑	❑	❑	❑	❑	❑	❑
Researching	❑	❑	❑	❑	❑	❑	❑
Teaching / coaching / workshop leading / mentoring	❑	❑	❑	❑	❑	❑	❑
Other 1	❑	❑	❑	❑	❑	❑	❑
Other 2	❑	❑	❑	❑	❑	❑	❑

4.5 We are interested in how you **normally** do each of these activities.

*For **each** activity, please pick the participation option that represents what you have **mainly** done.*

*By **online** we mean, for example, social media, websites, apps, live streaming, online forums, pre-recorded videos, etc.*

*By **offline** we mean in person or at a venue.*

*By **with others** we mean others who work in the arts and cultural areas, rather than spectators, listeners, audience members etc.*

{Logic based on 4.2}	Online alone	Online with others	Offline alone	Offline with others
Appraising / assessing / evaluating / reviewing	❑	❑	❑	❑
Composing / choreographing / designing / making / writing	❑	❑	❑	❑
Conducting / directing / producing	❑	❑	❑	❑
Curating / editing / presenting / technical supporting	❑	❑	❑	❑
Managing / promoting	❑	❑	❑	❑
Performing	❑	❑	❑	❑
Researching	❑	❑	❑	❑
Teaching / coaching / workshop leading / mentoring	❑	❑	❑	❑
Other 1	❑	❑	❑	❑
Other 2	❑	❑	❑	❑

4.6 Has **the current public health situation** affected how you have undertaken these activities.

*For **each** activity, please pick the participation option that represents what you have **mainly** done.*

{Logic based on 4.2}	Online alone	Online with others	Offline alone	Offline with others
Appraising / assessing / evaluating / reviewing	❑	❑	❑	❑
Composing / choreographing / designing / making / writing	❑	❑	❑	❑
Conducting / directing / producing	❑	❑	❑	❑
Curating / editing / presenting / technical supporting	❑	❑	❑	❑
Managing / promoting	❑	❑	❑	❑
Performing	❑	❑	❑	❑
Researching	❑	❑	❑	❑
Teaching / coaching / workshop leading / mentoring	❑	❑	❑	❑
Other 1	❑	❑	❑	❑
Other 2	❑	❑	❑	❑

4.7 Overall, **since the start of the current public health situation**, how often you have been able to **maintain your skills** as an artist, performer, maker etc.?

	I didn't do it	Much less	Quite a lot less	A lot less	No change	A little more	Quite a lot more	Much more
Learning / practising / preparing / reflecting **individually**	❑	❑	❑	❑	❑	❑	❑	❑
Learning / practising / preparing / reflecting **with others in person**	❑	❑	❑	❑	❑	❑	❑	❑
Learning / practising / preparing / reflecting **with others online**	❑	❑	❑	❑	❑	❑	❑	❑

4.8 Reflecting on how often you have been able to **maintain your skills** since the start of the current public health situation, how do you expect this to change in the **next month**?

	I won't do it	Much less	Quite a lot less	A lot less	No change	A little more	Quite a lot more	Much more
Learning / practising / preparing / reflecting **individually**	❑	❑	❑	❑	❑	❑	❑	❑
Learning / practising / preparing / reflecting **with others in person**	❑	❑	❑	❑	❑	❑	❑	❑
Learning / practising / preparing / reflecting **with others online**	❑	❑	❑	❑	❑	❑	❑	❑

4.9 Please select the picture that best describes how connected to other people you feel right now.

{Source: Inclusion of Other in Self Scale}

4.10 Please select the picture that best describes how connected to other people **who work in the arts and cultural areas** you feel right now.

{Adaptation of: Inclusion of Other in Self Scale}

5. Income and support

5.1 Considering all of your work in the arts and cultural areas, are you predominantly...

Sum should add to 100%.

Freelance
{Slider | Units: percentage}

Employed
{Slider | Units: percentage}

5.2 What is your **household's income** from all sources over the last 12 months (i.e. before the current public health situation)?

Count income from every person included in the household. Include:

All earnings (include overtime, tips, bonuses, self-employment)
All pensions (include all student grants and bursaries but not loans)
All benefits and tax credits (such as child benefit, income support or pension credit)
All interest from savings or investments
All rent from property (after expenses)
Other income (such as maintenance or grants)

Do not deduct taxes, National Insurance contributions, Health Insurance payments, Superannuation payments.

Numbers refer to your local currency.

❑ Up to £5,199
❑ £5,200 and up to £10,399
❑ £10,400 and up to £15,599
❑ £15,600 and up to £20,799
❑ £20,800 and up to £25,999
❑ £26,000 and up to £31,199
❑ £31,200 and up to £36,399
❑ £36,400 and up to £41,599
❑ £41,600 and up to £46,799
❑ £46,800 and up to £51,999
❑ £52,000 and up to £75,999
❑ £76,000 and above
❑ Would rather not say

5.3 Bearing in mind your answer above, what is **your individual contribution** to your household's income **from all sources (artistic and otherwise)** over the last 12 months (i.e. before the current public health situation)?

Do not deduct taxes, National Insurance contributions, Health Insurance payments, Superannuation payments.

{Slider | Units: percentage}

5.4 Approximately, how much of this (i.e. **your individual contribution** to household income) was directly from your **work in the arts and cultural areas** over the last 12 months (i.e. before the current public health situation)?

Do not deduct taxes, National Insurance contributions, Health Insurance payments, Superannuation payments.

{Slider | Units: percentage}

5.5 Approximately what percentage of your income came from each of the following areas in the **last 12 months** (i.e. before the current public health situation)?

Sum should add to 100%.

{Logic based on 4.2}

Appraising / assessing / evaluating / reviewing
{Slider | Units: percentage}

Composing / choreographing / designing / making / writing
{Slider | Units: percentage}

Conducting / directing / producing
{Slider | Units: percentage}

Curating / editing / presenting / technical supporting
{Slider | Units: percentage}

Managing / promoting
{Slider | Units: percentage}

Performing
{Slider | Units: percentage}

Researching
{Slider | Units: percentage}

Teaching / coaching / workshop leading / mentoring
{Slider | Units: percentage}

Other 1 (please specify) ___________________________
{Slider | Units: percentage}

Other 2 (please specify) ___________________________
{Slider | Units: percentage}

5.6 How much has your income changed in each of the following areas **since the start of the current public health situation**?

{Logic based on 4.2}

Appraising / assessing / evaluating / reviewing
{Slider | Units: £ | Range: (Lost -£5000 or more) – (Made £5000 or more)}

Composing / choreographing / designing / making / writing
{Slider | Units: £ | Range: (Lost -£5000 or more) – (Made £5000 or more)}

Conducting / directing / producing
{Slider | Units: £ | Range: (Lost -£5000 or more) – (Made £5000 or more)}

Curating / editing / presenting / technical supporting
{Slider | Units: £ | Range: (Lost -£5000 or more) – (Made £5000 or more)}

Managing / promoting
{Slider | Units: £ | Range: (Lost -£5000 or more) – (Made £5000 or more)}

Performing
{Slider | Units: £ | Range: (Lost -£5000 or more) – (Made £5000 or more)}

Researching
{Slider | Units: £ | Range: (Lost -£5000 or more) – (Made £5000 or more)}

Teaching / coaching / workshop leading / mentoring
{Slider | Units: £ | Range: (Lost -£5000 or more) – (Made £5000 or more)}

Other 1 (please specify) ___________________________
{Slider | Units: £ | Range: (Lost -£5000 or more) – (Made £5000 or more)}

Other 2 (please specify) ___________________________
{Slider | Units: £ | Range: (Lost -£5000 or more) – (Made £5000 or more)}

5.7 Do you consider yourself to be in financial hardship as a result of the current public health situation?

❑ No
❑ Yes, a little
❑ Yes, a lot

If yes

5.8 If you have lost income since the start of the current public health situation, are you able to recuperate it? (please tick all that apply)

❑ No, and I won't be able to
❑ Not yet, but I hope to **in full**
❑ Not yet, but I hope to **in part**
❑ Yes, through other modes of delivery (e.g. online teaching, live streaming) **in full**
❑ Yes, through other modes of delivery (e.g. online teaching, live streaming) **in part**
❑ Yes, through my employer(s) / contractor(s) **in full**
❑ Yes, through my employer(s) / contractor(s) **in part**
❑ Yes, through government-based initiatives **in full**
❑ Yes, through government-based initiatives **in part**
❑ Yes, other (please specify) ____________________

5.9 Since the start of the current public health situation, have you turned to others for information, advice, and support on **financial matters**?

❑ Yes
❑ No

If yes

5.10 **Where** have you turned for information, advice, and support on **financial matters**? (please tick all that apply)

❑ Colleague(s)
❑ Charities **(arts-specific)** / community-based organization(s)
❑ Charities **(general)** / community-based organization(s)
❑ Educational institution(s)
❑ Employer(s)
❑ Family / friend(s)
❑ Finance professional(s) **(arts-specific)** / advisor(s) / bank(s) / finance helpline(s)
❑ Finance professional(s) **(general)** / advisor(s) / bank(s) / finance helpline(s)
❑ Government-based agency(-ies)
❑ Insurer(s)
❑ Teacher(s) / coach(es) / mentor(s)
❑ Trade union(s) / professional body(-ies)
❑ Other (please specify) ______________________________
❑ Other (please specify) ______________________________

If yes and if no

5.11 Have you considered turning to any of the following people / places for any information, advice, and support on **financial matters**? (please tick all that apply)

❑ Colleague(s)
❑ Charities **(arts-specific)** / community-based organization(s)
❑ Charities **(general)** / community-based organization(s)
❑ Educational institution(s)
❑ Employer(s)
❑ Family / friend(s)
❑ Finance professional(s) **(arts-specific)** / advisor(s) / bank(s) / finance helpline(s)
❑ Finance professional(s) **(general)** / advisor(s) / bank(s) / finance helpline(s)
❑ Government-based agency(-ies)
❑ Insurer(s)
❑ Teacher(s) / coach(es) / mentor(s)
❑ Trade union(s) / professional body(-ies)
❑ Other (please specify) ________________________________
❑ Other (please specify) ________________________________

5.12 Since the start of the current public health situation, have you turned to others for information, advice, and support on **health and wellbeing matters**?

❑ Yes
❑ No

If yes

5.13 **Where** have you turned for information, advice, and support on **health and wellbeing matters**? (please tick all that apply)

❑ Colleague(s)
❑ Charities **(arts-specific)** / community-based organization(s)
❑ Charities **(general)** / community-based organization(s)
❑ Educational institution(s)
❑ Employer(s)
❑ Family / friend(s)
❑ Government-based agency(-ies)
❑ Health professional(s) **(arts-specific)** / advisor(s) / hospital(s) / health helpline(s)
❑ Health professional(s) **(general)** / advisor(s) / hospital(s) / health helpline(s)
❑ Insurer(s)
❑ Teacher(s) / coach(es) / mentor(s)
❑ Trade union(s) / professional body(-ies)
❑ Other (please specify) ________________________________
❑ Other (please specify) ________________________________

If yes and if no

5.14 Have you considered turning to any of the following people / places for any information, advice, and support on **health and wellbeing matters**? (please tick all that apply)

❑ Colleague(s)
❑ Charities **(arts-specific)** / community-based organization(s)
❑ Charities **(general)** / community-based organization(s)
❑ Educational institution(s)
❑ Employer(s)
❑ Family / friend(s)
❑ Government-based agency(-ies)
❑ Health professional(s) **(arts-specific)** / advisor(s) / hospital(s) / health helpline(s)
❑ Health professional(s) **(general)** / advisor(s) / hospital(s) / health helpline(s)
❑ Insurer(s)
❑ Teacher(s) / coach(es) / mentor(s)
❑ Trade union(s) / professional body(-ies)
❑ Other (please specify) ______________________________
❑ Other (please specify) ______________________________

6. Open response

In this section we are seeking your views, in your own words, of how the current public health situation has impacted on you and your work. Please write in as much detail as you can, including stories, examples and anecdotes.

6.1 How has the current public health situation impacted on **your arts and cultural work**? We are interested in any challenges and/or opportunities that you may be experiencing in relation to your work.

____

____

____

____

6.2 How has the current public health situation impacted on **your health and wellbeing**? We are interested in any challenges and/or opportunities that you may be experiencing in relation to your health and wellbeing.

____

____

____

____

7. Health and wellbeing

You are almost at the end of the survey!

We now have some questions we would like you to answer on your wellbeing.

7.1 Mental Health Continuum Short Form 14-item scale

7.2 Centre for Epidemiologic Studies Depression (CES-D) Short Form 8-item scale

We now have some questions we would like you to answer on your general health.

7.3 How is your health in general?

❑ Very good
❑ Good
❑ Fair
❑ Bad
❑ Very bad
❑ Would rather not say

7.4 Do you have any ongoing (chronic) health issues?

❑ No
❑ Yes
❑ Would rather not say

If yes

7.5 Do you consider yourself as someone living with...? (please tick all that apply)

❑ Mental health issues
❑ Cancer
❑ Cardiovascular disease
❑ Chronic pain
❑ Chronic respiratory diseases
❑ Yes, other (please specify)_________________

7.6 Physical activity scale in the Whitehall II Study

7.7 Overall, in the last month, has the public health situation affected how often you have undertaken **sports or other energetic activities** (e.g. walking, dancing, running)?

❑ Much less often
❑ Quite a lot less often
❑ A little less often
❑ No change
❑ A little more often
❑ Quite a lot more often
❑ Much more often

7.8 In the last month, has the public health situation affected how you have **mainly** done **sports or other energetic activities** (e.g. walking, dancing, running)?

*By **online** we mean, for example, social media, websites, apps, live streaming, online forums, pre-recorded videos, etc.*

*By **offline** we mean in person or at a venue.*

- ❑ I didn’t do any sports or other energetic activities
- ❑ Online alone
- ❑ Online with others
- ❑ Offline alone
- ❑ Offline with others

8. Social connections

Very nearly there. Keep going!

Please answer a few questions on your connections to other people.

8.1 Social Connectedness Revised 15-item scale

8.2 UCLA Three-Item Loneliness Scale, Single item loneliness question

8.3 De Jong Gierveld Loneliness Short Form 6-item scale

8.4 How often do you feel lonely?

- ❑ Always
- ❑ Often
- ❑ Sometimes
- ❑ Occasionally
- ❑ Hardly ever
- ❑ Never

8.8 How intense is this feeling?

- ❑ Not intense at all
- ❑ A little intense
- ❑ Neutral
- ❑ Quite intense
- ❑ Very intense

9. Further information

Thank you for your help!

If you have been affected by any of the issues in this survey, please see the sources of support section below.

We will be following-up this survey in couple of months to find out more about the impact of the current public health situation on the work, livelihood, and wellbeing of arts and cultural workers.

9.1 If you did not leave your email address on page 1 but wish to participate in the follow-up survey, please leave your email address here: _________________________

9.2 Please re-enter your email address: _________________________

Sources of support:

It is important to seek help from your GP or family doctor if you feel unhappy, sad, or anxious, or if the thought of harming yourself or others has occurred to you. If you think there's a danger of imminent harm to you or others call your local A&E services and ask to speak to the duty psychiatrist.

You can find information and support regarding wellbeing and mental health at the following links: NHS - Five steps to mental wellbeing; How to deal with stress; NHS - Stress and anxiety companion; Samaritans; Mind.

This NHS link provides information and processes for seeking support related to Coronavirus (COVID-19).

If you have any questions regarding this survey please contact our research team at hearts@rcm.ac.uk. More information about the study can be found here.
